# ExoOrb: A novel visual and analytical system for therapeutic extracellular vesicles metrics

**DOI:** 10.1016/j.csbj.2025.11.038

**Published:** 2025-11-19

**Authors:** Touseef Ur Rehman, Muhammad Rameez Ur Rahman, Weihua Tang, Sebastiano Vascon, Pei Jiang, Yu Liu, Senyi Gong, Xun Wan, Ali Mohsin, Meijin Guo

**Affiliations:** a State Key Laboratory of Bioreactor Engineering, East China University of Science and Technology, Shanghai 200237, PR China; b Department of Environmental Sciences, Informatics and Statistics Scientific campus, Ca' Foscari University of Venice, Via Torino, 155, Venezia, Mestre 30170, Italy; c Shanghai Morimatsu Pharmaceutical Equipment Engineering Co Ltd., No. 1340 Qianhui Road, Pudong, Shanghai, PR China

**Keywords:** Extracellular vesicles (EVs), Plant-derived EVs (PDEVs), Anticancer therapeutics, Multi-criteria decision making, ExoOrb

## Abstract

Extracellular vesicles (EVs) are naturally secreted nanoscale mediators of intercellular communication, showing potential for therapeutic and functional food applications. Although many EVs are being isolated with claims of therapeutic benefits, the evaluation criteria require extensive resources and time, often resulting in futile outcomes. This work addresses this gap by developing a visual and quantitative system using monk fruit cell-derived EVs (MFEVs) as a model to efficiently select the most suitable therapeutic EVs by analyzing their characterization parameters. This approach saves valuable resources and time. To generate variations, MFEVs were isolated using eight different techniques: ultracentrifugation, ultrafiltration, polyethylene glycol (PEG) precipitation (8 %, 10 %, 15 %, and 20 %), anion-exchange chromatography, and a novel combined ultrafiltration-precipitation method. Following isolation, their physicochemical properties, biochemical composition, and bioactivity were characterized, and their dose-dependent anticancer effects were evaluated across multiple cancer cell lines. Next, using data from the correlative statistics of anticancer activity with characterization parameters, “ExoOrb” is developed. It is an analytical multicriteria decision-making system that objectively ranks the therapeutic potential of EVs by employing factor normalization, weighted scoring, and multidimensional visualizations. The system has been validated using both the original dataset and synthetic datasets. The original dataset identified PEG 10 %-MFEVs as more effective therapeutically, and the synthetic dataset confirmed ExoOrb's ability for metrisizing EVs across multiple EVs types. To our knowledge, ExoOrb is the first potentially universal framework for evaluating the therapeutic potential of EVs based on characterization parameters, providing a reliable tool for scientific and therapeutic research through standardized, data-driven optimization.

## Graphical Abstract

**Figure:**
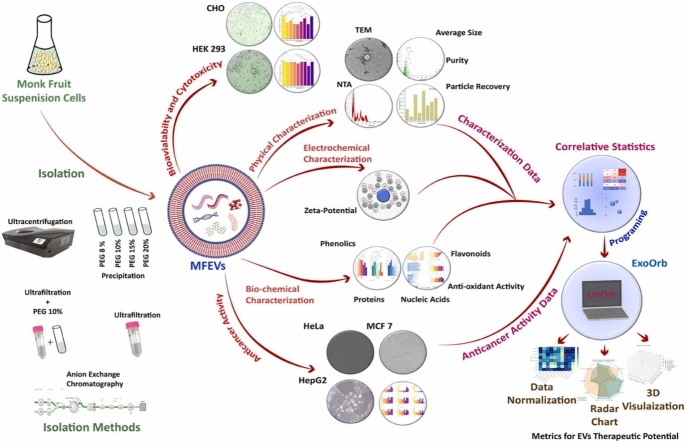

## 1 Introduction

Cellular organelles are fundamental biochemical and bioinformatic units that maintain cellular homeostasis [1]. Certain membrane-bound organelles are selectively released into the extracellular environment through regulated biological processes [2]. Indeed, extracellular vesicle (EV) secretion represents a universal biological phenomenon observed across all living cell types [3]. Structurally, EVs are nano-sized and are enclosed in a lipid bilayer containing biological components (proteins, nucleic acids, lipids) identical to the parent cell [4], [5]; they exhibit a spherical morphology [6]. These extracellular vesicles (EVs) function as critical mediators of intercellular communication, facilitating the transfer of biomolecules between cells [7].

Building on the fundamental biophysical properties of extracellular vesicles (EVs), plant-derived EVs (PDEVs), particularly those derived from traditional Chinese herbs and medicinal plants, exhibit remarkable therapeutic potential for multiple therapeutic conditions [8]. This bioactivity stems from their unique cargo of phytoactive biocomponents [9], which are capable of eliciting immunomodulatory responses [4]. At the same time, their conserved lipid bilayer structure enables inter-kingdom communication through biomolecular transfer [9]. Recent advances have highlighted PDEVs as promising candidates for both therapeutic and nutraceutical applications, owing to their oral bioavailability and recognition as safe, which confer distinct advantages over synthetic delivery systems [10]. Based on their established therapeutic potential, PDEVs from medicinal plants, such as aloe, lemon, ginger, turmeric, grapes, strawberries, and grapefruit, exhibit dual therapeutic roles. Beyond serving as natural nanocarriers for drug delivery, these vesicles possess intrinsic bioactive properties, making them promising candidates for treating neurological disorders and cancer [11]. Emerging research indicates that PDEVs can modulate gastrointestinal homeostasis through multiple mechanisms: (i) direct interaction with gut microbiota composition, (ii) attenuation of inflammatory pathways in intestinal tissue, and (iii) subsequent enhancement of both digestive function and systemic immune regulation [12]. Furthermore, PDEVs demonstrate dual anticancer advantages: (i) therapeutic activity mediated by tumor-suppressive miRNAs, angiogenesis inhibitors, and apoptotic proteins, and (ii) inherent safety via selective tumor targeting and antioxidant protection of normal tissues [13], [14]. This favorable biocompatibility and their inherent bioactivity position PDEVs as versatile therapeutic platforms for a wide range of medical conditions, including cancers [15].

While extracellular vesicle (EV) secretion occurs naturally in cellular physiology, their application in medical therapies and research requires standardized isolation protocols to ensure purity and functionality [16]. Current EV isolation strategies employ diverse techniques, including polymer-based precipitation, differential ultracentrifugation, and ultrafiltration to extract EVs from biological fluids and cell culture supernatants [17]. As EV research progresses, novel methodologies continue to emerge, emphasizing cost-effective, high-yield purification approaches [18]. Among available EV isolation methods, techniques such as size-exclusion chromatography and immunoaffinity capture, along with commercial kits, offer distinct advantages for preserving vesicle integrity during purification [19]. Whilst the isolation methodology critically determines key EV characteristics, including yield, purity, and biomolecular composition [20]. Consequently, rigorous post-isolation characterization is essential for both research and clinical applications. This typically involves multimodal analysis, combining biochemical profiling (e.g., protein and nucleic acid content) with biophysical assessments of particle size distribution, morphology, and surface charge (Zeta potential measurements) [21], [22]. EVs have been reported to contain various essential metabolites, including alcohols, sugars, carboxylic acids, amino acids, amides, and enzymes [23]. Additionally, EVs carry numerous nucleic acids and a diverse array of cellular proteins. Several protein biomarkers, such as CD63 and syntenin-1, have been identified as universal markers for EV characterization [24].

Meanwhile, adopted Isolation methods can affect EVs' therapeutic utility [25]. Differential ultracentrifugation remains the most widely adopted approach due to its scalability for large sample volumes, yet it suffers from low yield and potential vesicle damage [26]. Ultrafiltration provides superior particle recovery and purity [27]. Polymer-based precipitation reduces the purity with co-isolated contaminants [28]. These studies signify the fact that these methodological variations demonstrably influence EV physicochemical properties [20]. These findings demonstrate that using a single source while employing different isolation methods can introduce experimental noise, necessitating a standardized, multi-parametric approach that is crucial for benchmarking the therapeutic potential of EVs, encompassing metrics such as yield, purity, functionality, and biomolecular integrity.

Henceforth, we established monk fruit (*Siraitia grosvenorii*) cell-derived EVs (MFEVs) as a model system to develop a multicriteria decision-making analytical framework for the selection of optimal EVs with enhanced anticancer therapeutic potential. This perennial Cucurbitaceae vine, indigenous to China, possesses remarkable therapeutic potential due to its unique bioactive constituents [29] with traditional applications spanning respiratory (cough, cold) and gastrointestinal (constipation) disorders [30]. Recent phytochemical analyses have identified 15 therapeutic polyphenols in monk fruit cell cultures, including kaempferol-3-O-Glc-7-O-Rha and coumaroyl quinic acid derivatives, which exhibit dose-dependent antioxidant capacity [31], [32]. Backed by the therapeutic efficacy of monk fruit, this study systematically examines the changes in physicochemical characteristics of MFEVs using multiple isolation methodologies, complemented by their application in various mammalian cell lines. After evaluating the anticancer therapeutic potential of MFEVs in various cancer cell lines, quantitative reverse transcription polymerase chain reaction (qPCR) analysis was performed, revealing that MFEVs significantly increase the expression of the Bcl-2-associated X protein gene (BAX). It is well established that the upregulation of BAX is associated with the induction of oxidative stress, ultimately leading to apoptosis [33]. Furthermore, a reactive oxygen species (ROS) assay and apoptosis assays confirmed that MFEVs specifically induce this anticancer effect across all selected cancer cell lines by promoting oxidative stress, which results in the induction of apoptosis. After confirming the extraordinary anticancer ability of MFEVs, the interrelationships between their physical, electrochemical, and biochemical attributes and anticancer activity were analyzed through correlation. While correlation results exposed an intrinsic optimization conflict in physicochemical characterization-based anticancer therapeutic potential. Although all the EVs were from the same source, due to differences in adopted isolation techniques, the isolated EVs exhibited comparable variability in all characterization parameters; however, all the isolated EVs presented exceptional anticancer potential. Compared to prior studies [34], some EVs fell within the ideal size range, while others exhibited extraordinary Zeta potential. Similar variability was observed in other characterization parameters as well. Conversely, a number of isolated EVs exhibited some suboptimal characterization features, presenting a conflicting challenge. To resolve this challenge, we developed “ExoOrb”, a statistically advanced multi-criteria decision-making system, which enables data-driven selection of optimal anticancer EVs based on weighted performance metrics. ExoOrb addresses critical challenges in EV research by (i) quantitatively evaluating trade-offs between competing parameters and (ii) generating intuitive visual outputs that map the optimization landscape. This dual functionality enables researchers to objectively identify the best-fit EVs for anticancer applications by optimally balancing yield, purity, and functionality requirements. As a versatile analytical platform, ExoOrb provides transformative utility across extracellular vesicle research, from comparative analysis to therapeutic development.

## 2 Materials and methods

All experiments were rigorously optimized to achieve robust and reproducible results with statistical significance, utilizing triplicate measurements to account for both biological and technical variability. We implemented established peer-reviewed protocols with integrated controls to ensure assay consistency. Methodological rigor was further enhanced through: (i) use of calibrated instruments, (ii) verified reagent quality control, and (iii) maintenance of standardized environmental conditions during the study. Furthermore, wherever applicable, MFEVs are being presented with an isolation method name for easy differentiation and understanding.

### 2.1 Plant cell culture

*Siraitia grosvenorii* (*S. grosvenorii*) cell suspension cultures (Fig. S5) used in this study were obtained and established from cotyledons [31]. 50 g (wet cell weight) of *S. grosvenorii* callus cells were inoculated into 500 mL culture flasks containing 200 mL Gamborg’s B5 (B5) medium, which was elicited by adding 4 mg⋅L^−1^ 1-naphthylacetic acid (NAA), 0.2 mg⋅L^−1^ 6-benzylaminopurine (6-BA), and 30 g⋅L^−1^ sucrose. The pH of the suspension medium was kept at 5.8. Additionally, the culture media were sterilized at 121˚C for 25 min in an autoclave. The culture establishment was carried out in a sterilized environment, which was subsequently set up for growth on a rotary orbital shaker at 115 rpm and 25˚C under dark conditions. Subsurface sterilization was ensured by using 70 % ethanol. These cell suspension cultures were used for EVs isolation on the 7th day after inoculation.

### 2.2 CHO and HEK-293 cell cultures

Chinese hamster ovary (CHO) cells and HEK-293 cells (Yuchi Shanghai Biotechnology Co., Ltd., China) were cultured in GacTCHO β and Uni293 Expression medium (Shanghai Abiosys Biotechnology, China), respectively, and supplemented with 10 % fetal bovine serum (FBS, ExCell Bio), along with 100 U.mL^−1^ penicillin and 100 μg mL^−1^ streptomycin (Gibco, USA), which were incubated at 37 °C in a 5.0 % CO_2_ atmosphere to promote proliferation. Before performing cytotoxic analysis, cells were counted using a Countstar (Shanghai RuiYu Biotech Co., Ltd., China), and 5000 cells were inoculated into each well of 96-well plates. These cells were incubated for 24 h at 37 °C and a 5.0 % CO_2_ atmosphere before initiation of cytotoxic analysis. For bio-dispersal and bio-availability analysis, 10,000 cells were inoculated into each well of 6-well plates before the initiation of bio-dispersal and bio-availability analysis.

### 2.3 Cancer Cell Cultures

HepG2 cells (Yuchi Shanghai Biotechnology Co., Ltd., China) were cultured according to the previously described method by Yan et al. [35]. Specifically, HepG2 cell cultures were established in RPMI-1640 (CelHappy, JSBio, China) supplemented with 10 % fetal bovine serum (FBS, ExCell Bio, China), 100 U.mL^−1^ penicillin, and 100 μg.mL^−1^ streptomycin (Gibco, USA). While HeLa cells (Yuchi Shanghai Biotechnology Co., Ltd., China) were cultured according to Mathieu et al. [36]. Cell cultures were established in DMEM/High glucose (CelHappy, JSBio, China), which was further supplemented with 10 % fetal bovine serum (FBS, ExCell Bio, China) along with 100 U.mL^−1^ penicillin and 100 μg.mL^−1^ streptomycin (Gibco, USA). MCF-7 cells (Yuchi Shanghai Biotechnology Co., Ltd., China) were cultured accordingly to Song et al. [37], *i.e.,* cell cultures were established in DMEM/High glucose (CelHappy, JSBio, China) by adding 10 % fetal bovine serum (FBS, ExCell Bio, China), which was supplemented with 100 U.mL^−1^ penicillin and 100 μg.mL^−1^ streptomycin (Gibco, USA) along with 10 μg.mL^−1^ of human recombinant insulin (Shanghai Basalmedia Technologies, China). The established cancer cell cultures were maintained for further proliferation at 37°C in a 5.0 % CO_2_ atmosphere. Before performing cytotoxic analysis, cells were counted using CountStar (Shanghai RuiYu Biotech Co., Ltd., China). 5000 cells were inoculated/well in 96-well plates for cytotoxicity analysis and reactive oxygen species assay, while 100,000 cells/cell line were used in 6-well plates for qPCR. The cultures were incubated for 24 h at 37 °C and 5.0 % CO_2_ atmosphere before initiation of cytotoxic analysis.

### 2.4 MFEVs isolation

**Fig. 1:**
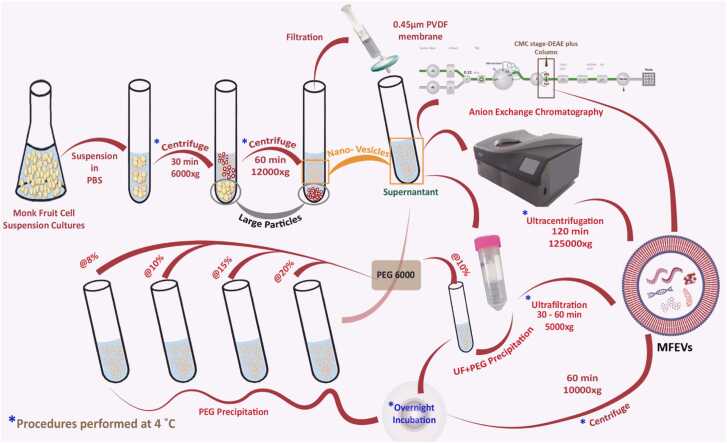
Steps adopted for isolation of MFEVs in different Isolation Methods.

Multiple methods were used to analyze the methodological impact of isolation methods on the therapeutic potential of MFEVs from cell suspension cultures (Fig. 1). For the preparation of supernatant, cells from the suspension culture were separated from the suspension solution and resuspended in PBS pH 7.4 before centrifugation at 6000 × g for 30 min, followed by re-centrifugation at 12000 × g for 1 h at 4.0˚C in order to remove large particles. Further, the supernatant was filtered through a 0.45 µm PVDF membrane (Millipore, USA). From the final supernatant, 40 mL of the supernatant/isolation run was used to isolate MFEVs further using different methods. The particle concentration of the supernatant was recorded by NTA (Fig. S1).

#### 2.4.1 Ultracentrifugation (UC)

The method was adapted with g-force adjustment according to the Minimal Information for Studies of Extracellular Vesicles guideline [38]. Ultracentrifugation was performed by using Beckman´s Coulter OPTIMA MAX-XP Ultracentrifuge, USA, at 125000 × g for 2 h at 4.0˚C. The resulting pellets were collected, washed, resuspended, and then diluted to 5 mL in PBS (pH 7.4).

#### 2.4.2 Precipitation (P)

The method for precipitation-based isolation was adapted from Kalarikkal et al. [39], in detail, 8, 10, 15, and 20 % of PEG 6000 was used (weight/volume), after the addition of PEG supernatant solutions containing PEG concentrations were incubated overnight at 4.0˚C followed by centrifugation at 10000 × g for 1.0 h at 4.0˚C, resulting MFEVs were collected, washed, resuspended, and then diluted to 5 mL in PBS (pH 7.4).

#### 2.4.3 Ultrafiltration (UF)

For ultrafiltration, the Amicon® Ultra 100 K device (Millipore, USA) was used according to the manufacturer's protocol. The supernatant was filtered using an ultrafiltration device and then centrifuged at 5000 × g for 1 h at 4.0 °C.

#### 2.4.4 Ultrafiltration Followed by PEG Precipitation (UF+PEG 10 %)

The supernatant was added to the Amicon Ultra 100 K device and centrifuged at 5000 × g for 1 h. In the resulting solution, PEG 6000 @ 10 % was added (weight/volume); after addition of PEG, supernatant solutions containing PEG were incubated overnight at 4.0˚C, which were later differentially centrifuged at 10000 × g for 1 h at 4.0˚C, resulting MFEVS were collected, washed, resuspended, and diluted to 5 mL volume in PBS pH 7.4.

#### 2.4.5 Anion Exchange Chromatography (AEC)

The procedure for anion exchange chromatography was adapted from the method reported by Heath et al. [40] and aligned with the manufacturer's protocol for the column used. The 1 mL CMC stage-DEAE plus Monolithic Column (YU JI (Shanghai) Biological Technology Co., Ltd., China) was balanced by using buffer marked as A, containing 50 mM HEPES, 50 mM NaCl, 1.0 % Sorbitol, and 0.05 % poloxamer (Polyoxy ethylene oxypropene), at pH 7.5. The supernatant was loaded into the column at a rate of 3 mL.min^−1^ using an ÄKTA explorer chromatography system (Amersham Biosciences, Sweden). After sample loading, the column was re-washed with Buffer A, and eluted with Buffer B (50 mM HEPES and 2.0 M NaCl) at 3 mL.min^−1^ to release MFEVS. The resulting solution was concentrated to 5 mL volume using an Amicon® Ultra 100 K device at 4.0˚C.

### 2.5 Post-isolation analysis and storage

For short-term storage, EVs were suspended in PBS (pH 7.4) and preserved at −80°C, with all analyses completed within 4–6 weeks post-isolation to maintain sample integrity

### 2.6 Characterization of MFEVs

For transmission electron microscopy (TEM) analysis, the samples were placed on copper grids coated with carbon. The grids were dried to remove excess solution, followed by negative staining with 2 % (w/v) aqueous uranyl acetate. After further drying, MFEVs samples from different isolation methods (UC, P (PEG 8–20 %), UF, and UF+PEG 10 %) were analyzed on an HT 7700 TEM (Hitachi, Japan), whereas MFEVs from AEC were analyzed on a JEM-1400 (JEOL, Japan) (TEM). To determine the MFEV's concentration and size, nano tracking analysis (NTA) was performed using a Nanosight NS300 (Malvern, UK), while Zeta potential was measured with a Zetasizer Pro-Blue (Malvern, UK).

#### 2.6.1 MFEVs particles recovery

The universal percentage calculation method determined the particle recovery percentage, i.e., the comparison of the total particle yield from each isolation method based on NTA with the total particles present in the supernatant. The equation used for calculating particle recovery percentage is as follows:

$$\mathrm{Particle Recovery}\%=[\frac{\left\{\mathrm{NTA}\left(\mathrm{Exosomes}\right)\times \mathrm{Total Volume}\left(\mathrm{Exosomes}\right)\right\}}{\left\{\mathrm{NTA}\left(\mathrm{Supernatant}\right)\times \mathrm{Total Volume}\left(\mathrm{Supernatant}\right)\right\}}]\;\times 100$$

#### 2.6.2 MFEVs total nucleic acids quantification (TNC)

Total nucleic acid quantification was performed on the NanoDrop 2000 (ThermoFisher Scientific, USA), and samples were analyzed with and without lysis. Lysis was performed with lysis buffer (MagMAX plant RNA isolation kit, ThermoFisher Scientific, USA) as per the prescribed protocol without any nucleic acid degradation, and total nucleic acids were separated from magnetic beads with Nuclease-free water. Furthermore, for analysis, 5 µL of each sample was pipetted into the Nanodrop, and the reading was recorded using the manufacturer-provided software. Analysis was performed in triplicate; data were recorded, analyzed, and compared using the following formula to quantify the internal nucleic acid content of MFEVs.

Iexn=Texn−Rexn

Where *Iexn* is MFEVs' internal nucleic acids concentration, *Texn* is MFEVs' total nucleic acids concentration, and *Rexn* is MFEVs' residual nucleic acids concentration. *Texn* values were calculated after the lysis of MFEVs, whereas *Rexn* values were calculated without the lysis of MFEVs.

#### 2.6.3 Proteins analysis of MFEVs

##### 2.6.3.1 Protein concentration analysis (PC) for MFEVs

For protein concentration studies, analyses were performed to determine total protein, protein outside MFEVs, and protein inside MFEVs. Specifically, protein concentration was determined using the BCA protein assay kit, with absorbance measured at 595 nm according to the manufacturer's protocol (Beyotime Biotech Inc., China). In this analysis, protein concentration was analyzed with and without RIPA lysis. Analysis was performed in triplicate; data were recorded, analyzed, and compared using the following formula to calculate the internal protein content of MFEVs.

Iexp=Texp−Eexp

Where *I*exp  is MFEVs' internal protein content, *T*exp  is MFEVs' total protein content, and *E*exp  is MFEVs' external protein content. *T*exp  was calculated after RIPA lysis of MFEVs, whereas *E*exp  was calculated without lysis of MFEVs.

##### 2.6.3.2 SDS-PAGE analysis for MFEVS

To perform the SDS-PAGE, samples containing MFEVS were ultrasonically crushed and lysed with RIPA lysis buffer according to the manufacturer's protocol (Beyotime Biotech Inc., China). Monk fruit cells were also ultrasonically crushed, followed by RIPA lysis buffer for SDS-PAGE. In order to check, differentiate, and identify bands of proteins inside MFEVs, a single sample from UF-MFEVs was analyzed without RIPA lysis. Furthermore, SDS-PAGE was performed using a 12.5 % Fast SDS-PAGE kit (Shanghai EpiZyme Biotechnology Co., Ltd., China) according to the manufacturer's protocol, with a 15–150 kDa protein ladder used.

##### 2.6.3.3 Western blot analysis for MFEVS

After lysis with RIPA buffer, the samples were run through a 15 % Fast SDS-PAGE kit, manufactured by Shanghai EpiZyme Biotechnology Co., Ltd., China, according to the manufacturer's protocol. After electrophoresis, the proteins were transferred to a PVDF membrane, followed by blocking with 5.0 % milk. The membranes were later incubated with primary antibodies anti-syntenin, CD63 (EVs positive marker), and calnexin (negative/contaminant marker) (all from Abcam, UK) at 4.0˚C for 24 h. Following washes, membranes were incubated with the HRP-conjugated secondary antibody, goat anti-rabbit IgG (Signalway antibody, China), for 1 h. Detection was performed using an Enhanced Chemiluminescence (ECL) substrate (EasyBlot ECL kit, Sangon Biotech Shanghai Co., Ltd., China) and visualized using a chemiluminescence imaging system (Tanon, China).

#### 2.6.4 MFEVs purity

Purity based on proteins of MFEVS from each method was calculated as the average particle concentration ratio to the average protein concentration. For calculating MFEVS purity, the external protein content (*E*exp ) was used, as it represents the protein concentration without RIPA lysis. Furthermore, the purity based on nucleic acids was calculated by the average particle concentration ratio and the average residual nucleic acid concentration (*Rexn).*

#### 2.6.5 Total phenolics content (TPC) in MFEVs

Total phenolic content was measured by the Folin-Ciocalteu assay [41] with adjustments. Briefly, 20 µL of each MFEVs sample was taken in triplicate. After adding 100 µL of methanol, the mixture was vortexed for 10 min and then centrifuged at 10,000 × g for 5 min. 500 µL of distilled water was added to every 120 µL mixture. Later, 125 µL of Folin-Ciocalteu reagent was added to the resulting mixture and incubated for 6 min in complete darkness. Then, 1.25 mL of 7.0 % Na_2_CO_3_ was added and incubated for 90 min in the dark. Finally, absorbance at 765 nm was recorded. The catechol standard curve was used for the interpretation of results.

#### 2.6.6 Total flavonoid content (TFC) in MFEVs

Total flavonoid content was measured by the aluminum chloride colorimetric assay [42] with adjustments; 125 µL of MFEVs samples was added to 500 µL of methanol, followed by the addition of 37.5 µL NaNO_2_ (1.0 mol.L^−1^). The mixture was then vortexed and incubated for 3.0 min. Subsequently, 37.5 µL of AlCl3 (10 %) (w/v) was added, vortexed again, and incubated for an additional 3.0 min. This was followed by the addition of 250 µL NaOH (1.0 mol.L^−1^), and was vortexed and incubated in the dark for 40 min. Absorbance was measured at 510 nm, and the whole procedure was performed in triplicate. Furthermore, the Rutin standard curve was used for the final quantification of total flavonoids.

#### 2.6.7 Anti-oxidant activity (DPPH) of MFEVs

For analyzing the antioxidant activity of MFEVs, the 1,1-diphenyl-2-picrylhydrazyl (DPPH) assay [39] with adjustments. 20 µL of each MFEVS sample was mixed with 100 µL of ethanol and vortexed for 10 min. The mixture was then centrifuged at 10000 × g for 5.0 min. 7.89 mg of DPPH reagent was dissolved in 100 mL of ethanol to form the DPPH reagent solution, which was incubated in the dark for 2 h. A 100 µL sample extract was added to 900 µL of DPPH reagent solution (DRS), followed by incubation for 30 min at room temperature in complete darkness. The DPPH reagent solution alone served as a control. The absorbance was checked at 517 nm by using a UV spectrophotometer. Whereas the antioxidant activity of the sample was calculated as

$$\mathrm{Antioxidant Activity}=[\frac{\mathrm{Control}A-\mathrm{Sample}A}{\mathrm{Control}A}]\;\times 100$$

*Control A* is the absorbance of the (DRS) control. Whereas, Sample A is the absorbance of each sample.

### 2.7 MFEVs Bio-dispersal and Bio-availability in normal cells

UF-MFEVs were stained with a green fluorescence Hieff® Exosomes tracker kit (Yeasen Biotech Company, Shanghai, China) according to the manufacturer’s protocol prior to application to HEK-293 and CHO cells. 20 µL stained UF-MFEVs were applied to HEK-293 and CHO cell cultures in 6-well plates and were incubated for 24 h at 37 °C and a 5.0 % CO_2_ atmosphere. Furthermore, bio-dispersal and bio-availability were assessed using a Mshot Fluorescence microscope (Guangzhou Micro-shot Technology Co., Ltd., China), and images were captured at 2, 6, 12, and 24 h using the MShot Image Analysis System.

### 2.8 MFEVs induced cell cytotoxicity analysis

Cell viability and cytotoxicity were evaluated using the CCK8 assay (Shanghai Aladdin Biochemical Technology Co., Ltd., China) across various normal and cancerous cell lines, following the manufacturer's protocol for analysis. In detail, the reinoculated cells were transferred to 96-well plates, and the culture media were carefully removed. Fresh culture media containing 1 × 10^9^, 1 × 10^10^, and 1 × 10^11^ MFEV particles/mL from each isolation method were added, respectively. For the apoptosis assay on cancer cell lines, 1 × 10^10^ particles/mL of UF-MFEVs was utilized. On the other hand, cells treated with fresh culture media containing no MFEVs served as a control, and cultures were further incubated for 24 h at 37 °C in a 5.0 % CO_2_ atmosphere. After 24 h, 10 µL of CCK-8 solution was added to each well and further incubated for 5 h before measuring the absorbance at 450 nm. Furthermore, for the detection of bioavailability in cancer cell lines, PEG10 % MFEVs were stained with a red fluorescence Hieff® Exosomes tracker kit (Yeasen Biotech Company, Shanghai, China) according to the manufacturer’s protocol, and images were captured at 2, 6, 12, and 24 h. In contrast, apoptosis detection by fluorescence was performed with Annexin V-FITC (Beyotime Biotechnology, Shanghai) according to the manufacturer’s protocol. Visualizations were performed by a Mshot Fluorescence microscope (Guangzhou Micro-shot Technology Co., Ltd., China), and images were taken by the MShot Image Analysis System.

### 2.9 MFEVs induced intracellular reactive oxygen species (ROS) detection

Intracellular reactive oxygen species (ROS) were detected in multiple cancer cell lines using the ROS Assay Kit (Beyotime Biotechnology, Shanghai, China). Following cell stabilization, cells were treated with UF-MFEVs 1 × 10^10^ particles/mL in fresh medium for 24 h. Control cells received fresh medium only. After 24 h, the positive control wells were treated with Rosup for an additional 25 min to induce ROS. All cells were then incubated with the fluorescent probe DCFH-DA for 20 min. Following three washes with serum-free medium, the resulting fluorescence signal, indicating intracellular ROS, was immediately visualized using a fluorescence microscope.

### 2.10 Quantitative real-time polymerase chain reaction (qPCR)

To analyze the expression of the BAX gene, total RNA was first isolated from cancer cells (treated with UF-MFEVS at 1 × 10^10^ particles/mL or untreated control) using an RNA extraction kit (Beyotime Biotechnology, Shanghai, China). The purified RNA was immediately reverse-transcribed into complementary DNA (cDNA), including a genomic DNA (gDNA) removal step, using the ABScript III RT Master Mix for qPCR with gDNA Remover (ABclonal, Wuhan, China). qPCR was then performed using the synthesized cDNA, the Taq Pro Universal SYBR qPCR Master Mix (Vazyme, Nanjing, China), and gene-specific primers (Beyotime Biotechnology, Shanghai, China). The relative expression of BAX was determined by normalizing the data to the expression of the housekeeping gene, β-actin (Human).

### 2.11 MFEVs anticancer correlative statistics

A Pearson correlation was performed between cancer cell viability percentages and all physicochemical factors related to MFEVs to identify relationships among variables using Origin Pro, and the results were analyzed accordingly.

### 2.12 ExoOrb

We introduce ExoOrb, a multi-criteria decision-making system [43] to compare different EVs based on MFEVs' anticancer correlative statistical data. ExoOrb offers a statistical and interpretable approach to comparing and selecting the most suitable EVs for anticancer use. The development of ExoOrb involves the following steps.

#### 2.12.1 Factor normalization

In ExoOrb, the initial step is factor normalization, which ensures comparability across different factors. Factors are divided into maximization and minimization based on the direction in which they contribute to the anticancer therapeutic potential of MFEVs. Specifically, these categories reflect whether higher values (maximization) or lower values (minimization) of each factor are desirable. For maximization factors, such as particle recovery and MFEVs purity, normalization is performed by dividing the value for each method by the maximum value of that factor. The normalized value $N_{\mathrm{ij}}^{\max}$ for a factor j and method i is given by:

$$N_{\mathrm{ij}}^{\max}=\frac{X_{\mathrm{ij}}}{\max\left(X_{j}\right)}$$

where $X_{\mathrm{ij}}$ represents the original value of factor j for method i, and $\max\left(X_{j}\right)$ is the maximum value of the factor j across all methods.

For minimization factors, such as average size and TPC, normalization is performed by dividing the minimum value of each factor by the value for each method. The normalized value $N_{\mathrm{ij}}^{\min}$ for a factor j and method i is calculated as:

$$N_{\mathrm{ij}}^{\min}=\frac{\min\left(X_{j}\right)}{X_{\mathrm{ij}}}$$

where $\min\left(X_{j}\right)$ represents the minimum value of the factor j across all methods.

For the special case of Zeta potential, where values may be negative, the data is first shifted by adding 100 to make all values positive before applying the normalization. The value 100 is chosen according to the Zeta potentials’ range set as (-100 to +50) [44]. The shifted values are normalized in a manner similar to other minimization factors. This ensures that negative values are handled appropriately without distorting the results.

#### 2.12.2 Weighted score calculation

Once the factors are normalized, we calculate a weighted score for each EV, taking into account the relative importance of each factor. Let $w_{j}$ be the weight assigned to the factor j, where $w_{j}\in \left[0,1\right)$ and $\sum_{j=1}^{n}w_{j}=1$. The overall weighted score $S_{i}$ for method i is computed as:

$$S_{i}=\sum_{j=1}^{n}w_{j}\cdot N_{\mathrm{ij}}$$

where $N_{\mathrm{ij}}$ is the normalized value of factor j for method i, and n is the total number of selected factors.

This weighted sum aggregates the performance across all factors for each EVs, ensuring that factors with higher weights contribute more significantly to the final score. Higher scores reflect better overall performance based on the selected criteria. In our implementation, we assigned equal weights to EV factors isolated from each method, as correlation analysis showed that all the considered factors contributed substantially to the anticancer potential of MFEVs. To test the model's sensitivity and stability, we performed an analysis in which different weights were intentionally assigned to the parameters.

#### 2.12.3 Ranking of EVs anticancer therapeutics

After calculating the weighted scores, the EVs are ranked according to their scores, with higher scores corresponding to better rankings. The rank $R_{i}$ of EVs i is assigned based on the descending order of the weighted scores:

$$R_{i}=\text{rank}\left(S_{i}\right)$$

EVs with the highest score are assigned rank 1, indicating the best overall performance.

### 2.13 ExoOrb validation using real and synthetic data sets

Firstly, the real data set (Supplementary file 2), derived from the characterization of all isolated MFEVs, was analyzed to rank and evaluate different EV formulations. This analysis was necessary because the use of diverse isolation methods introduced sufficient experimental noise at the characterization level. Secondly, randomized computational datasets simulating (i) 15 EV types with 11 characterization parameters (Supplementary file 3), and (ii) 8 EV types with 13 characterization factors (including physicochemical properties, functional properties, and practical factors)(Supplementary file 4). Data distributions were modeled using Gaussian curves for continuous variables (*e.g*., Zeta potential) and Poisson distributions (*e.g*., particle count), with Monte Carlo simulations introducing experimental noise [45]. Parameter correlations (*e.g*., yield-purity trade-offs) were maintained to reflect real-world trends. Most importantly, the synthetic data sets contained values relatable to data from various EV studies [19], [34], [46], [47], [48]. These synthetic datasets were analyzed using ExoOrb to objectively compare EV characterization factors and demonstrate ExoOrb's ability to identify the best-fit EVs in anticancer applications, reflecting optimization trade-offs, and to validate ExoOrb’s decision algorithms in silico (Table S2).

### 2.14 Software

Python was used for ExoOrb development. Images were created with Adobe Illustrator, graphs were generated with OriginPro®, and the manuscript was prepared in Microsoft Word.

### 2.15 Statistical analysis

All data were analyzed using OriginPro, employing one-way ANOVA followed by Tukey's honestly significant difference (HSD) post-hoc test, and Pearson correlation to assess relationships between different parameters where applicable. A p < 0.05 was considered statistically significant. The significance levels are indicated as * p < =0.05, ** p < =0.01, and *** p < =0.001.1.

## 3 Results

### 3.1 Characterization of MFEVs

**Fig. 2:**
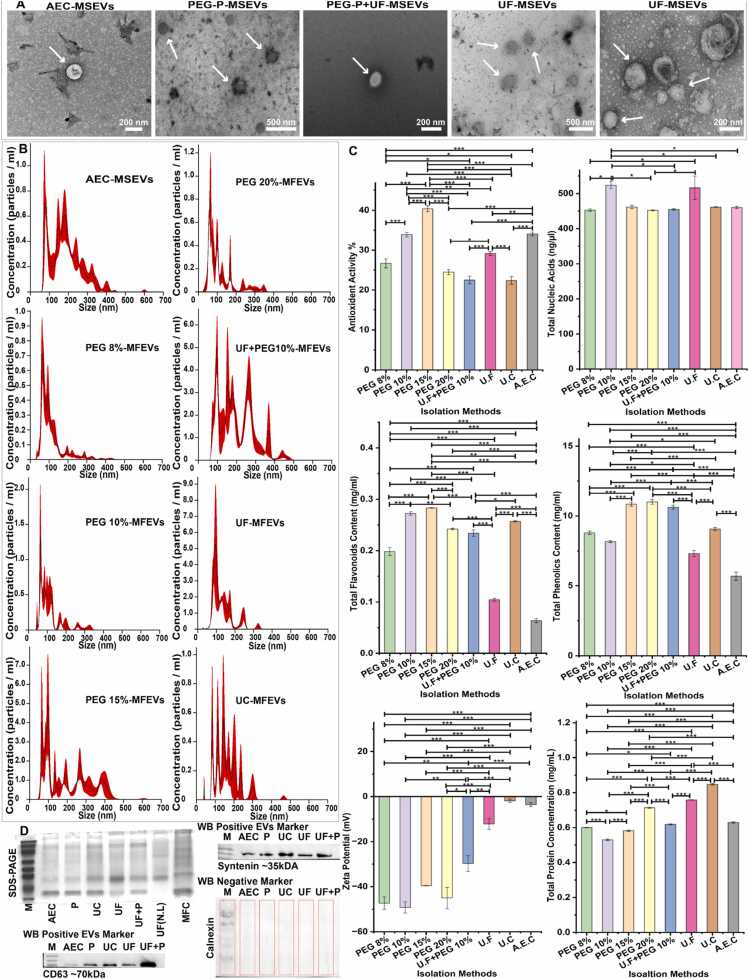
Characterization of MFEVs. **(A)** MFEVs TEM images; **(B)** NTA particle concentration; **(C)** Electrochemical, biochemical, and biofunctional characterization; **(D)** SDS-Page and WB (M represents marker and AEC (anion exchange chromatography), P (precipitation PEG 10 %), UC (ultracentrifugation), UF (ultrafiltration), UF+P (ultrafiltration followed by PEG 10 % precipitation), UF (N.L) (ultrafiltered MFEVS where no RIPA lysis was performed), MFC (Monk fruit cells).

TEM analysis (Fig. 2A) is crucial for assessing the morphology of EVs [49], which confirmed the membrane-bound, spherical-to-ovoid morphology of MFEVs, with structural variations observed across MFEVs isolated by different isolation methods. Crucially, method-dependent morphological variations were observed, particularly in the integrity of vesicle membranes and aggregation patterns. NTA data (Fig. 2B) revealed three key findings: (i) UF+PEG 10 % combination method EVs achieved 2–3-fold higher particle yields than conventional UC, (ii) AEC-MFEVs generated the highest absolute particle concentrations, (iii) UF+PEG 10 % uniquely excluded sub-50 nm particulates, reducing potential contaminants as compared to PEG precipitation alone.

Size distribution profiles (Table S1) demonstrated that all isolation methods produced MFEVs within the average size range (110–200 nm), with > 80 % of particles below 300 nm (Fig. S2), which is critical for EVs biodistribution efficiency [50]. Particle recovery rates are a critical metric for isolation efficiency [51] Meanwhile, AEC isolated MFEVs achieved the highest particle recovery rate, followed by UF-MFEVs, while the conventional UC-MFEVs presented minimal recovery (Table S1).

Electrochemical properties are very important for nanoparticles’ therapeutic applications [52]. Zeta potential measurements (Fig. 2C) revealed substantial variation in surface charge (−1.8 to −49.12 mV), with PEG-based EV isolation methods clustering at the maximum. This charge consistency signifies PEG’s efficiency for EVs' surface properties, potentially enhancing stability [53].

Total nucleic acid content varied substantially between isolation methods (Fig. 2C), with variability in nucleic acid levels inside and outside MFEVs (Fig. S4) providing insight into protocol stringency influences EV cargo integrity.

Protein characterization revealed that UC-MFEVs contained the highest protein content (Fig. 2C) and exhibited the highest external protein content (Fig. S3). However, PEG had an inverse influence on the external protein content of MFEVs (Fig. S3). While SDS-PAGE identified lysis-sensitive bands (20–25 and 70–100 kDa), suggesting intra-EVs proteins (Fig. 2D). Furthermore, Western blot confirmation of CD63 and syntenin-1 validated EV identity across all methods, whereas membranes incubated with negative contaminant marker antibody calnexin visualized no band intensity, indicating trustworthy purity across all isolated MFEVs (Fig. 2D).

EVs' purity assessment is crucial [50]. While isolation methods can influence the EVs' purity based on proteins and nucleic acids [54]. Computationally, MFEVs with PEG 8 % presented the highest purity regarding proteins, while AEC led in nucleic acid purity (Table S1). Importantly, MFEVs isolated using all methods fall within the purity range: 1.034E+ 10–2.899E+ 11 for proteins and 8.63514E+ 10–1.2695E+ 12 for nucleic acids, which further signifies their suitability for downstream applications.

Phytochemical analysis revealed isolation-dependent retention of bioactive compounds. TPC peaked in PEG 20 % isolates (Fig. 2C), and TFC (Fig. 2C), though lower than phenolics, were highest in PEG 15 % isolates, with enhanced antioxidant activity (Fig. 2C), presenting a trend of flavonoid-dependent antioxidant activity [55] suggesting isolation method-specific presence and activity of these metabolites.

### 3.2 Cancer cell cytotoxicity analysis of MFEVs

#### 3.2.1 Anticancer activity of MFEVs against HepG2

**Fig. 3:**
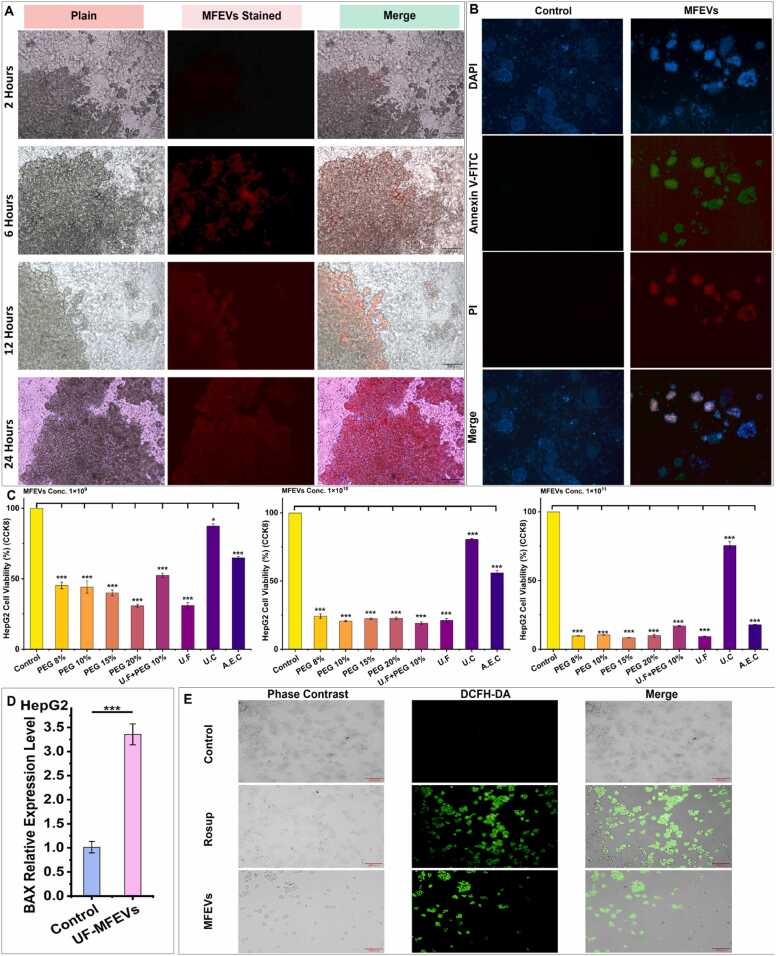
MFEVS anticancer activity in the HepG2 cell line**. (A)** Bio-dispersal and bioavailability of MFEVs to HepG2 cell line; **(B)** MFEVS induced apoptosis in HepG2 cancer cells; **(C)** MFEVs-induced dose-dependent toxicity in HepG2 cancer; **(D)** BAX relative expression level after MFEVs administration; **(E)** MFEVs induced ROS activity in HepG2 cells.

MFEVs demonstrated dose-dependent cytotoxicity against HepG2 cells, with all tested concentrations (1 × 10^9^, 1 × 10^10^, and 1 × 10^11^ particles.mL^−1^) significantly inhibiting proliferation compared to the controls. The anticancer activity intensified with increasing MFEVs concentrations, and efficacy varied across isolation methods (Fig. 3C). MFEVs exhibited rapid bioavailability, dispersing uniformly in culture within 24 h (Fig. 3A).

While apoptosis assays signified the induction of apoptosis specifically by MFEVs administration in the HepG2 cell line (Fig. 3B). The ROS activity assay provided evidence of a significant increase in oxidative stress post MFEVs administration specifically (Fig. 3E). These MFEVs induced anticancer effects are further supported by a highly significant upregulation of the BAX gene, which is well-known for associating with oxidative stress induction (Fig. 3D).

#### 3.2.2 Anticancer Activity of MFEVs Against HeLa

**Fig. 4:**
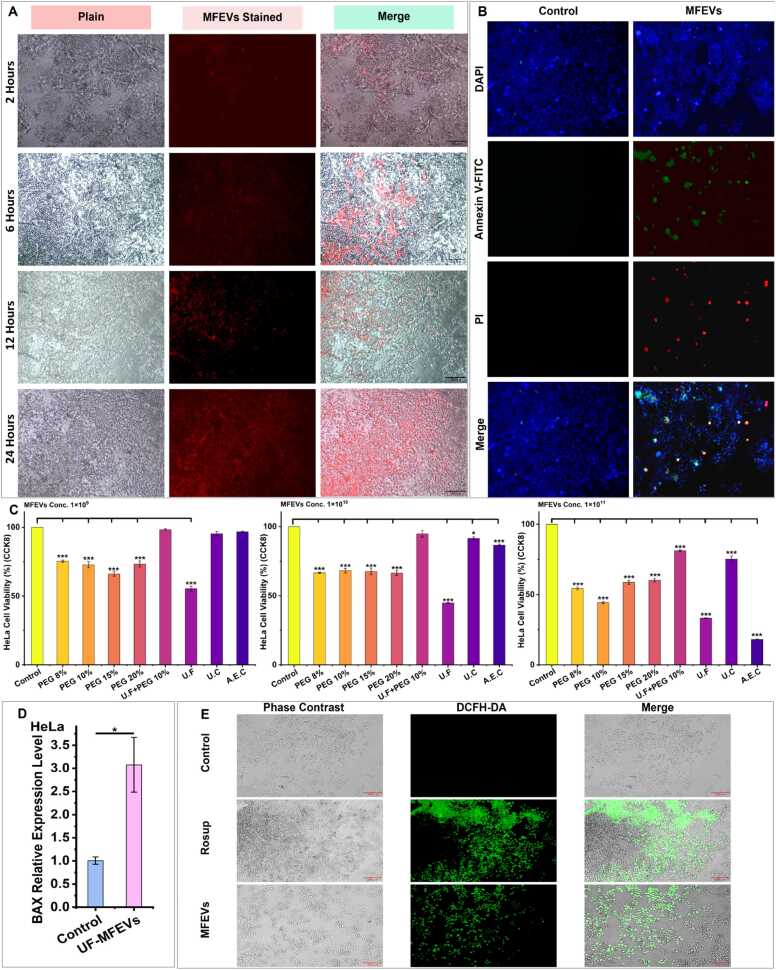
MFEVS anticancer activity in the HeLa cell line. **(A)** Bio-dispersal and bioavailability of MFEVs to HeLa cell line; **(B)** MFEVs-induced apoptosis in HeLa cell line; (C) Dose-dependent MFEVs-induced toxicity in HeLa cancer cells; **(D)** Post-MFEVs administration BAX relative expression level in HeLa cells; **(E)** MFEVs-specific ROS induction in HeLa Cells.

MFEVs exhibited isolation method- and concentration-dependent cytotoxicity against HeLa cells (Fig. 4C). At 1 × 10^9^ particles.mL^−1^, only MFEVs isolated via UF+PEG10 %, UC, and AEC showed no significant cytotoxicity, whereas PEG precipitation- and UF-derived MFEVs significantly reduced viability. This trend persisted at 1 × 10^10^ particles.mL^−1^, with UF+PEG 10 % remaining non-cytotoxic. At a concentration of 1 × 10^11^ particles.mL^−1^, all isolation methods induced significant cell death, demonstrating dose-responsive activity. Notably, MFEVs exhibited rapid bioavailability, uniformly dispersing in culture within 24 h (Fig. 4A). While apoptosis assays confirmed the induction of programmed cell death (Fig. 4B), this result corroborated the cytotoxicity findings.

These findings highlight that (i) MFEVs' anticancer effects intensify with particle concentration, (ii) MFEVs' anticancer efficacy varies by isolation protocol. Furthermore, MFEVs administration led to a significant increase in oxidative stress, as detected by the ROS activity assay (Fig. 4E). The observed anticancer effects are corroborated by the significant upregulation of the BAX gene (Fig. 4D), which links the MFEVs' action directly to a mechanism involving oxidative stress induction.

#### 3.2.3 Anticancer Activity of MFEVS Against MCF-7

**Fig. 5:**
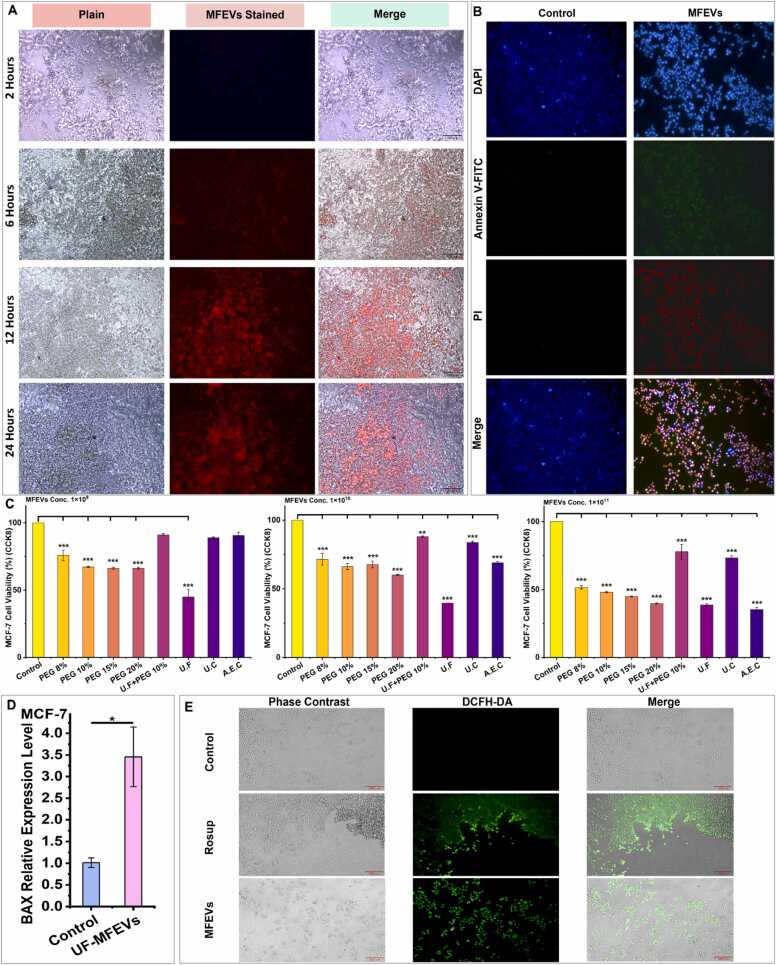
MFEVS induced anticancer activity in the MCF-7 cell line**. (A)** Bio-dispersal and bioavailability of MFEVS to MCF-7 Cell line; **(B)** MFEVS induced apoptosis in MCF-7 cell line; **(C)** MFEVs-induced dose-dependent toxicity in MCF-7 cancer cells; **(D)** BAX relative expression level following MFEVs application in MCF-7 cells; **(E)** MFEVs induced ROS activity in MCF-7 cells.

Building on the observed dose-dependent cytotoxicity against HeLa cells, MFEVs similarly demonstrated potent anticancer effects on MCF-7 breast cancer cells (Fig. 5C). The therapeutic impact varied significantly based on both isolation method and administered concentration: (i) At 1 × 10^9^ particles.mL^−1^: Only MFEVs prepared using UF+PEG 10 %, UC, and AEC methods showed negligible cytotoxicity, mirroring the pattern seen in HeLa cells. In contrast, PEG-precipitated and UF-isolated vesicles exhibited marked cell death induction. (ii) At higher concentrations (1 × 10^10^-1 × 10^11^ particles.mL^−1^): MFEVs from all isolation methods produced significant cytotoxic effects, with potency escalating proportionally to particle concentration, consistent with the HeLa cell results. The therapeutic potential was further supported by: (i) Rapid cellular uptake and uniform dispersion within 24 h (Fig. 5A) and (ii) Substantial MFEVs-specific apoptosis induction (Fig. 5B).

Henceforth, a molecular mechanism appears to support the anticancer action of MFEVs. This is evidenced by a significant surge in oxidative stress, as shown in the ROS activity assay (Fig. 5E), post-MFEVs administration. Furthermore, the significant upregulation of the BAX gene (Fig. 3D), a gene strongly associated with oxidative stress, provides corroborating support for the induction of MFEVs-specific anticancer effect.

### 3.3 MFEVs Bio-dispersal, bioavailability, and cytotoxicity in normal cells

**Fig. 6:**
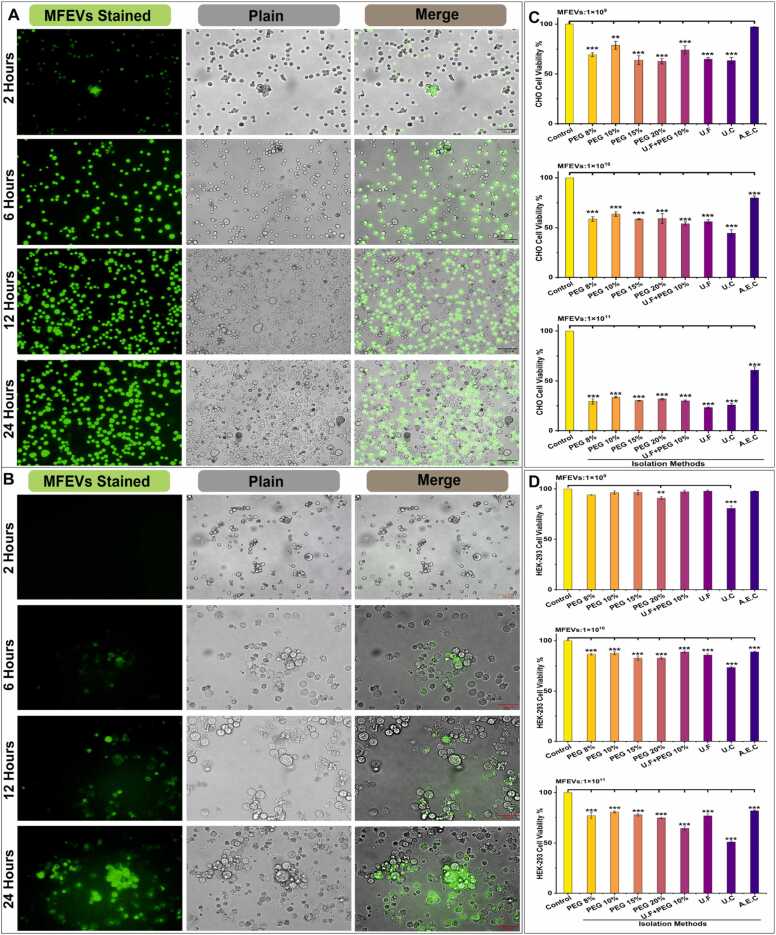
MFEV's application to CHO and HEK-293 cell lines. **(A)** MFEVs bioavailability to CHO cell line; **(B)** MFEVs bioavailability to HEK-293 cell line; **(C)** Dose-dependent MFEVs induced cytotoxicity to CHO; **(D)** Dose-dependent MFEVs induced cytotoxicity to HEK-293.

Visual microscopic analysis demonstrated distinct uptake kinetics between CHO and HEK-293 cells, where MFEVs showed rapid bioavailability in CHO cells (60.7 % within 2 h) that decreased to 48.5 % by 24 h despite proliferation, while HEK-293 cells exhibited delayed but progressive uptake (0 % at 2 h, 76.7 % at 6 h, peaking at 81.29 % by 24 h) (Fig. 6 A, B, and S6). Cytotoxicity assessments revealed concentration-dependent effects, with both cell lines maintaining viability above 90 % at biologically safe doses (≤ 1 × 10^9^ particles.mL^−1^). Notably, MFEV's isolation methods had a differential impact on cytotoxicity in both cell lines, suggesting cell-type-specific interactions related to MFEV's uptake and therapeutic effects (Fig. 6C and D).

### 3.4 MFEVs anticancer correlative statistics

**Fig. 7:**
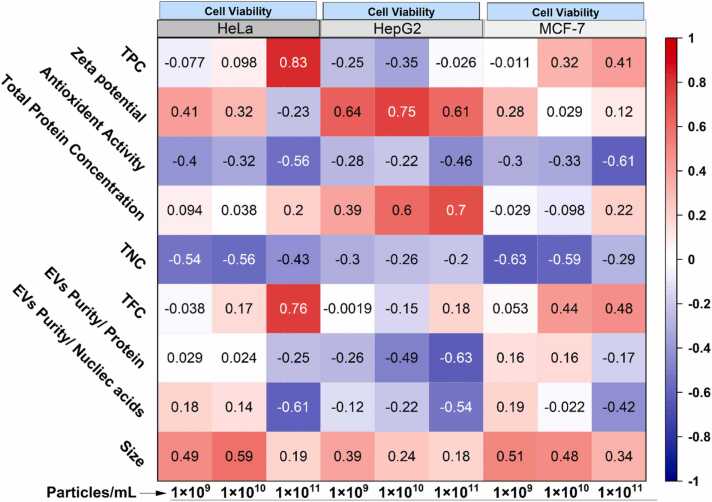
MFEVs' physicochemical characteristics and anticancer effects correlative statistics.

Pearson statistical correlation analysis (Fig. 7) revealed critical relationships between all MFEVs' characteristics and cancer cell viabilities, which varied by both MFEVs concentrations and targeted cell types. As MFEVs' concentrations increased from 1 × 10^9^–1 × 10^11^ particles.mL^−1^, we observed distinct patterns: TPC showed a strong positive correlation with HeLa and MCF-7 viability, but a negative correlation with HepG2, while TFC exhibited increasingly positive correlations across all cell lines at higher concentrations. Physical properties demonstrated consistent trends, *i.e*., vesicle size positively correlated with viability in all cases, whereas purity (protein/nucleic acid ratios) shifted from positive to negative correlations with increasing concentration. Surface charge measurements revealed cell-specific behaviors, with Zeta potential showing a strong positive correlation for HepG2 but a negative correlation for HeLa cells.

However, antioxidant activity was consistently negatively correlated with viability, whereas protein content exhibited mostly positive associations, particularly in HepG2 cells. Notably, nucleic acid content showed inverse relationships with viability in all cancer cell lines. These concentration-dependent and cell-type-specific correlations demonstrate that isolation methods have a significant influence on the physicochemical properties of MFEVs and their subsequent therapeutic potential. All these variabilities at the characterization level and cancer-targeting stage provide a concrete basis for creating experimental noise, which is crucial for developing a computational model [56]. The comprehensive dataset was subsequently integrated into ExoOrb.

### 3.5 Real-time EVs anticancer therapeutic metrisizing test with ExoOrb

**Fig. 8:**
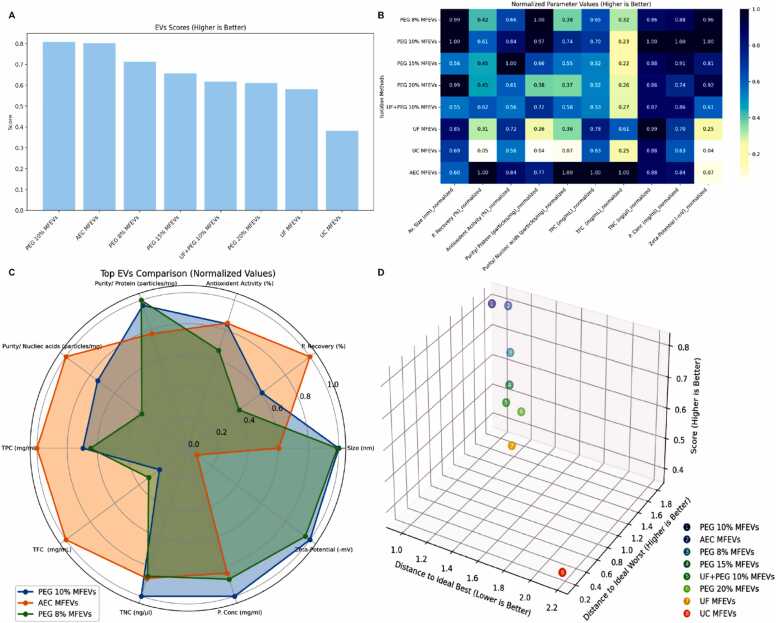
Real-time comparison and visualization of EVs' anticancer therapeutic potential in ExoOrb. (A) EVs' overall score; **(B)** Factor normalization; **(C)** Radar chart top 3 EVs factor-based comparison; **(D)** 3D visualization plot presenting the EVs potential therapeutic rankings.

As presented in the previous results, it is clear that although there is a significant increase in cytotoxicity against cancer cells after MFEVs are administered at increasing doses, it remains unclear which type of EVs is considerably more effective, as drug-induced cytotoxicity at all dosage concentrations is important [57]. Hence, the data set was organized and analyzed in ExoOrb, which immediately analyzed the data and yielded commendable results. The normalized data visualization (Fig. 8B) revealed EV-specific strengths. AEC-MFEVs outperformed others in particle recovery, nucleic acid-based purity, and bioactive content (TPC/TFC), whereas MFEVs from 8 % PEG excelled in protein purity. PEG 10 %-MFEVs dominated in vesicle size, Zeta potential, protein concentration, and total nucleic acid content, while MFEVs isolated by PEG 15 % showed peak antioxidant activity. While the bar chart visualization (Fig. 8A) presented the overall computed score strength of EVs with PEG 10 % domination, the radar chart further expands the visualization (Fig. 8C) to the overall effectiveness of MFEVs as PEG 10 % > AEC > PEG 8 %, with PEG 10 %-MFEVs covering the largest multivariate profile area with respect to physicochemical characterization factors. However, 3D decision-making visualization (Fig. 8D) demonstrated that while PEG 10 %-MFEVs currently lead, no method fully achieves the "ideal" solution of 100 %, highlighting the strength of ExoOrb in preventing overfitting, as overfitting in data analysis can be misleading [58]. This analysis quantified each EVs proximity to both best and worst case scenarios in real-time, providing a systematic computational framework for future EVs research.

#### 3.5.1 Synthetic data set analysis in ExoOrb

Through systematic analysis of synthetic datasets, ExoOrb has demonstrated its rigorous capability to quantitatively evaluate and rank EVs by assessing multiple characterization factors and their complex interrelationships. ExoOrb’s adaptable framework accommodates diverse parameters, including important EVs characteristics (size, concentration, purity), functional properties (biocompositional, antioxidant activity), and practical considerations (time, cost), with customizable optimization criteria for either maximization or minimization, and weightage allocation optimization for specific factors, based on research requirements. This flexibility establishes ExoOrb as a potentially universal analytical tool for comparative assessment of EVs aimed at therapeutics.

The platform's efficiency is exemplified by its streamlined one-click analysis interface (Video S1) and comprehensive data visualization outputs of synthetic data sets (Fig. S7 and S8), which enable (i) Multidimensional comparison of EVs performance across all evaluated parameters, (ii) Objective identification of optimal EVs for specific applications, and (iii) Data-driven optimization of EVs characteristics.

By integrating these capabilities, ExoOrb provides researchers with a powerful decision-support system that bridges the gaps for optimal anticancer EVs selection, ultimately facilitating the EVs research.

## 4 Discussion

This study presents a systematic investigation into the influence of various isolation methodologies on the therapeutic potential of MFEVs against multiple cancer cell lines. Through an integrated approach combining physical, electrochemical, and biochemical characterization with functional anticancer assays, we have gained critical insights into method-dependent variations in the properties and bioactivity of MFEVs. Culminating in the development of ExoOrb, an innovative platform for quantitative evaluation and selection of optimal anticancer EVs based on multi-parametric criteria, as discussed.

### 4.1 Novel multi-dimensional properties for qualitative and quantitative evaluation of MFEVs

The quality and therapeutic efficacy of extracellular vesicles are fundamentally determined by their physicochemical and biological properties [59]. While previous studies have examined individual characterization parameters such as size distribution [60], particle concentration [61], Zeta potential [62], and biochemical compositional analysis [63] in EV studies, this study provides an integrated insight into all these factors, including anticancer activity assessment. All the findings are corroborated by previous studies regarding EVs characterization and therapeutic applications. As demonstrated by Rasmussen et al., particle size and surface charge significantly influence the nanotherapeutic profiles of MFEVs [64]. Parallel phenomena with MFEVs are observed herein, where variations in these parameters across isolation methods resulted in evidently diverse cytotoxicity patterns against HepG2, HeLa, and MCF-7 cell lines. Results also revealed that PEG-containing isolation methods consistently produced MFEVs with superior Zeta potential values, aligning with established knowledge about PEG's effects [65]. The impact of isolation methodology on vesicle recovery and purity has been previously documented in mammalian EV studies [66]. This work extends this understanding to plant-derived EVs, demonstrating that the isolation technique correspondingly affects MFEVs' yield, purity, size distribution, and surface characteristics. Extending to MFEVs' anticancer potential, MFEVs' nucleic acid concentration presented a positive correlation for the induction of anticancer effect, as plant-derived nucleic acids have been considered to bear anticancer properties [67]. Moreover, MFEVs presented an exceptional anticancer effect in vitro, by inducing highly significant cytotoxicity in multiple cancer cell lines. Meanwhile, the apoptosis assay further supported the cytotoxicity analysis, as the assay's results showed MFEVs specifically induce apoptosis. Building upon these findings, the ROS activity assay demonstrated that MFEVs induce these cytotoxic effects through increased oxidative stress, with qPCR confirming MFEVs' significant ability to upregulate the BAX gene, a well-known factor in oxidative stress induction. While the correlational statistics between the characterization factors from each isolated MFEV type and their collateral anticancer effects uncovered relationships between the characterization factors and the anticancer potential. Based on the cumulative response of these relationships, ExoOrb, a computational multi-criteria decision-making system, was developed.

### 4.2 ExoOrb: multi-criterion analytical decision-making platform

The ExoOrb platform was developed to address the critical need for standardized evaluation of extracellular vesicles for anticancer applications, building upon our comprehensive analysis of how different techniques affect the characteristics and therapeutic potential of MFEVs. Unlike previous studies that examined isolated EVs, such as those from ultracentrifugation for purity [22], ultrafiltration for stability [68], or precipitation for yield [69]. This study integrates multiple evaluation criteria into a unified analytical framework by ExoOrb. This system quantitatively assesses EVs based on user-defined customizable parameters, including physical characteristics (size, concentration), electrochemical properties (Zeta potential), biochemical composition (protein, nucleic acid, phenolic content, flavonoid content), and antioxidant capacity.

Original and synthetic data-based validation demonstrated ExoOrb's ability to objectively rank EVs aimed for therapeutic application. For the original data set created in this study, the platform identified PEG 10 %-MFEVS as the optimal (score: 0.808), combining high particle recovery (51.8 %) with excellent colloidal stability (–49.12 mV) and balanced purity. While AEC-MFEVs closely followed (score: 0.801), particularly excelling in nucleic acid purity (85.4 % recovery). Furthermore, ExoOrb's analytical power is further demonstrated by its ability to identify performance trade-offs. While MFEVs isolated by hybrid methods, such as UF+PEG10 %, showed moderate overall scores (0.616), they exhibited specific advantages in particle recovery (53.0 %), which may be valuable for particular applications. Conversely, conventional UC-MFEVs scored poorly (0.381), with low recovery and instability (–1.87 mV), which aligns with the recently raised concerns about the limitations of UC-derived isolates [70]. Similarly, the analysis with synthetic data sets was also performed effectively, with reports generated (Supplementary files 5 and 6). Furthermore, ExoOrbs' sensitivity was also analyzed by varying the weightage for the parameter, revealing a critical insight (Fig. S9). While variability was observed among EVs lying in the intermediate panel, the most efficient EVs, *i.e.*, PEG10 %-MFEVs, consistently maintained their highest ranking; similarly, the least performing UC-MFEVs invariably remained at the bottom (Fig. S10). This stability in the model's extremes is a strong positive result, confirming that ExoOrb's core rankings are not susceptible to slight changes in weightage. This validates the tool's excellent ability to reliably metricize therapeutic EVs regardless of minor shifts in user-defined priorities.

With these validations, it's factual that the platform incorporates advanced visualization tools that transform complex multidimensional data into actionable insights. Radar charts (Fig. 8B) facilitate the immediate comparison of EVs' strengths across all evaluated parameters, while 3D modeling (Fig. 8C) effectively presents the proximity of EVs to ideal solutions. These visualizations not only confirm ExoOrbs' ability to overcome problems such as overfitting and underfitting but also reveal its capacity to find optimal solutions for therapeutic applications.

Furthermore, as compared to previously studied methods, ExoOrb's robustness stems from its foundation in our experimental findings, combined with established characterization parameters from the published literature. By incorporating known property-activity relationships, such as the association between nucleic acid content and enhanced anticancer effects, the platform advances beyond simple parameter measurement to provide scientifically grounded recommendations that yield superior outcomes, as demonstrated in Table 1.

**Table 1:** Comparison of ExoOrb with previously used approaches.

**Method**	**Employed For**	**Limitation**	**Reference**	**Superiority of ExoOrb**
Analytic network process	Nanoparticle preparation method selection	Mathematically straightforward but static	[71]	Aimed at EV therapeutics, which uses dynamic visualizations and enables customizable modifications of weights, turning a simple, static ranking into a transparent, interactive trade-off analysis.
Decision support system	Biomaterial safety assessment	Used logical rules and expert consensus to generate prioritized lists of assays needed for safety assessment	[72]	ExoOrb focuses on EV selection based on actual characterization parameters, not regulatory planning. It provides dynamic sensitivity analysis on multiple core trade-offs.
EVs purity benchmarking	EVs purity	Exhibit trade-off between purity and yield	[73]	Composite scoring that balances purity with other conflicting factors
Direct comparison	EVs isolation methods	Presenting raw values, overfitting ‘best method’ decision for the reader	[74]	Computational, considering multiple criteria, prevents overfitting and underfitting

This represents a significant advance over previous studies, offering researchers a decision-making tool with a framework for standardized EV therapeutic assessment while considering the complex interplay between isolation methodologies, vesicle characteristics, and therapeutic function. Previous studies have confirmed the credibility of data-driven methodologies for their potential for therapeutic and clinical applications [75]. Similarly, ExoOrb’s user-defined, customizable ability to add parameters, set maximization and minimization behaviors, and allocate customizable weights makes it a potentially universal multicriteria decision-making system for a wide range of scientific and therapeutic applications.

### 4.3 Future directions

In this study, ExoOrb is introduced as a potentially universal multi-criteria framework for selecting best-fit therapeutic EVs based on available characterization factors. However, further studies are necessary for in vivo proof of concept, analysis with multiple EV types, and integration with EV-omics databases like ExomiRHub, and web servers such as IMOPAC [76], [77]. These data integrations with omics databases will pointedly accelerate the therapeutic application of EVs [78].

## 5 Conclusion

This study clearly shows the method-dependent differences in the physicochemical properties and anticancer effectiveness of MFEVs, emphasizing the urgent need for standardized, multi-parameter evaluation. Our research not only clarifies the links between isolation methods, MFEVs characteristics, and bioactivity but also introduces ExoOrb. This innovative, multi-criteria analysis platform represents a significant advancement, offering the scientific community a measurable, robust, and customizable system for objective assessment, ranking, and selection of the most promising therapeutic EV candidates. ExoOrb effectively converts complex, multidimensional data into actionable insights, creating a new standard for EV-based nanotherapeutic standardization.

## CRediT authorship contribution statement

**Weihua Tang:** Project administration, Investigation, Formal analysis, Data curation. **Sebastiano Vascon:** Writing – review & editing, Supervision, Software, Formal analysis. **Touseef Ur Rehman:** Writing – review & editing, Writing – original draft, Visualization, Validation, Software, Methodology, Investigation, Formal analysis, Data curation, Conceptualization. **Muhammad Rameez Ur Rahman:** Visualization, Software, Investigation, Formal analysis, Data curation, Conceptualization. **Ali Mohsin:** Writing – review & editing, Validation, Supervision, Resources, Project administration, Conceptualization. **Meijin Guo:** Writing – review & editing, Validation, Supervision, Resources, Project administration, Methodology, Conceptualization. **Senyi Gong:** Formal analysis, Data curation. **Xun Wan:** Formal analysis, Data curation. **Pei Jiang:** Validation, Formal analysis, Data curation. **Yu Liu:** Formal analysis, Data curation.

## Consent for publication

Not applicable.

## Ethical statement

This research was conducted in accordance with internationally recognized ethical standards for animal-free research. With strict adherence to the 3Rs principles (Replacement, Reduction, Refinement), this study employed exclusively alternative methodologies, utilizing advanced in vitro systems with computational modeling approaches, minimizing the need for any animal experimentation. All human-derived biological materials, where applicable, were procured through ethically approved sources with appropriate institutional oversight and compliance with relevant biosecurity protocols. The authors affirm that this investigation is completely animal-free and declare no competing interests that could compromise the ethical integrity of this work.

## Declarations

**ExoOrb Code and Link**

https://github.com/ram95d/ExoOrb

https://exoorb-nidbmvccbsldhdq2taqpwh.streamlit.app/

## Funding

This work was financially supported by the National Foreign Expert Program of China (Grant number: Y20240198) and also by 10.13039/501100001809National Natural Science Foundation of China (Grant No. 22250410275).

## Declaration of Competing Interest

The authors have no competing financial or personal interests to declare.

## Data Availability

The data that support the ﬁndings of this study are available from the corresponding author upon reasonable request

## Footnotes

Appendix A Supplementary data associated with this article can be found in the online version at doi:10.1016/j.csbj.2025.11.038.
