# Supplementary material for: ExoOrb: A novel visual and analytical system for therapeutic extracellular vesicles metrics
Source: Comput Struct Biotechnol J. 2025 Nov 19;27:5289–306. doi: 10.1016/j.csbj.2025.11.038 (PMC12681852; doi:10.1016/j.csbj.2025.11.038)

| MFEVs | Average Size | Particle Recovery | Purity/Nucleic Acids | Purity/Proteins |
| --- | --- | --- | --- | --- |
|  | nm | % | particles/mg | particles/mg |
| PEG 8% | 111.2 | 35.78 | 4.78E+11 | 2.90E+11 |
| PEG 10% | 110.2 | 51.81 | 9.38E+11 | 2.80E+11 |
| PEG 15% | 197.5 | 38.36 | 7.02E+11 | 1.92E+11 |
| PEG 20% | 110.9 | 38.84 | 4.69E+11 | 1.09E+11 |
| UF+PEG 10% | 199.2 | 52.96 | 7.36E+11 | 2.08E+11 |
| Ultrafiltration | 130.0 | 26.53 | 4.56E+11 | 7.43E+10 |
| Ultracentrifugation | 159.8 | 4.06 | 8.64E+10 | 1.03E+10 |
| A.E.C | 183.2 | 85.40 | 1.27E+12 | 2.24E+11 |

**Supplementary Material**

**Table S1**. Representation of MFEVs characterization factors including Size Distribution, Recovery Efficiency, and Purity Assessment (Nucleic Acids & Proteins) with respect to adopted isolation.

**Table S2**. A complex synthetic data set for analysis in ExoOrb, the dataset includes multiple EVs with multiple factors.

**Data Set 1**

| **EVs** | **Time (hrs)** | **Zeta Potential (mV)** | **Yield (particles/mL)** | **Purity (EV:Protein)** | **Cost ($/sample)** | **Oxidative Stress (%)** | **Mean Size (nm)** | **RNA Yield (ng/10⁹ EVs)** | **Protein Contaminants (μg/mL)** | **Storage Stability (days)** | **Endotoxin (EU/mL)** | **Recovery Efficiency (%)** | **Throughput (samples/day)** |
| --- | --- | --- | --- | --- | --- | --- | --- | --- | --- | --- | --- | --- | --- |
| **MSC** | **6.2** | **-21.5** | **1.05E+10** | **1.91E+08** | **5800** | **71** | **155** | **5.2** | **55** | **6** | **1.1** | **82** | **8** |
| **hu-MSC** | **7.5** | **-25.8** | **3.10E+10** | **1.72E+09** | **3200** | **79** | **135** | **8.1** | **18** | **16** | **0.7** | **75** | **6** |
| **Apple** | **1.8** | **-25.2** | **1.02E+11** | **1.28E+10** | **2700** | **85** | **125** | **15.6** | **8** | **34** | **0.4** | **94** | **15** |
| **Orange** | **0.9** | **-14.7** | **7.20E+11** | **3.27E+09** | **650** | **53** | **220** | **2.8** | **220** | **4** | **4.5** | **65** | **40** |
| **Apple Cells** | **2.7** | **-30.3** | **5.10E+09** | **8.50E+08** | **8200** | **91** | **115** | **20.3** | **6** | **22** | **0.25** | **88** | **12** |
| **MSC (SEC)** | **1.2** | **-22.9** | **8.30E+10** | **5.53E+10** | **11500** | **87** | **120** | **25.1** | **1.5** | **32** | **0.15** | **97** | **5** |
| **Olive** | **3.3** | **-18.1** | **2.15E+09** | **4.78E+07** | **4800** | **68** | **150** | **10.4** | **45** | **15** | **0.65** | **79** | **25** |
| **Milk** | **4.2** | **-28.4** | **4.25E+10** | **2.83E+09** | **6000** | **80** | **125** | **18.7** | **15** | **28** | **0.4** | **85** | **10** |

**Data Set 2**

| **EVs** | **Time** | **Zeta-potential** | **Yield** | **Purity** | **Cost** | **Antioxidant** | **Size** | **RNA Yield** | **Protein Contaminants** | **Storage Stability** | **Endotoxin** |
| --- | --- | --- | --- | --- | --- | --- | --- | --- | --- | --- | --- |
| **Ginger** | **6** | **-20** | **1.00E+10** | **2.00E+08** | **6000** | **69** | **150** | **5** | **50** | **7** | **1.2** |
| **Garlic** | **7** | **-25** | **3.00E+10** | **1.50E+09** | **3000** | **77** | **130** | **8** | **20** | **14** | **0.8** |
| **MSC** | **1.5** | **-25** | **1.00E+11** | **1.00E+10** | **2500** | **82** | **120** | **15** | **10** | **30** | **0.5** |
| **hu-MSC** | **0.75** | **-15** | **5.00E+11** | **2.50E+09** | **500** | **58** | **200** | **2** | **200** | **3** | **5** |
| **Apple** | **2.5** | **-30** | **5.00E+09** | **1.00E+09** | **8000** | **88** | **110** | **20** | **5** | **21** | **0.3** |
| **Orange** | **3** | **-18** | **2.00E+11** | **5.00E+09** | **4500** | **65** | **140** | **10** | **40** | **14** | **0.7** |
| **Apple Cells** | **1** | **-22** | **8.00E+10** | **4.00E+10** | **10000** | **85** | **115** | **25** | **2** | **30** | **0.2** |
| **MSC (SEC)** | **2** | **-17** | **1.00E+11** | **1.67E+09** | **2000** | **60** | **160** | **6** | **60** | **10** | **1.5** |
| **Olive** | **1** | **-12** | **7.00E+11** | **4.67E+09** | **700** | **55** | **220** | **3** | **150** | **5** | **4** |
| **Tobacco** | **4** | **-28** | **4.00E+10** | **2.67E+09** | **6000** | **80** | **125** | **18** | **15** | **28** | **0.4** |
| **Milk** | **1.5** | **-35** | **6.00E+09** | **7.50E+08** | **3500** | **90** | **105** | **22** | **8** | **21** | **0.6** |
| **Buckwheat** | **2** | **-26** | **2.00E+10** | **8.00E+08** | **7000** | **78** | **130** | **12** | **25** | **14** | **0.9** |
| **Ginger (PEP)** | **1** | **-14** | **3.00E+11** | **3.00E+09** | **900** | **62** | **180** | **4** | **100** | **7** | **3** |
| **Garlic (PEP)** | **3.5** | **-20** | **2.00E+10** | **6.67E+08** | **1500** | **70** | **135** | **9** | **30** | **14** | **0.7** |
| **Rice leaves** | **1.5** | **-19** | **9.00E+10** | **1.13E+09** | **1200** | **68** | **155** | **7** | **80** | **10** | **2** |

**Figure S1.** Particle concentration and intensity in supernatant.


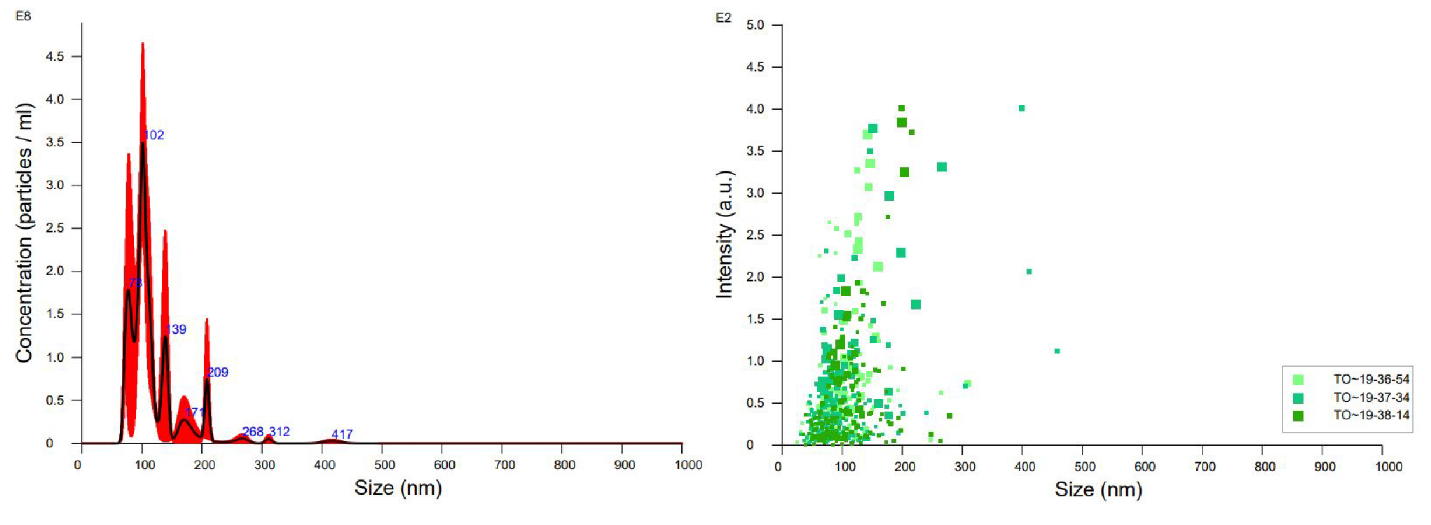


**Figure S2.** Particle intensity (a.u.) of MFEVs isolated from all the methods with respect to size distribution.


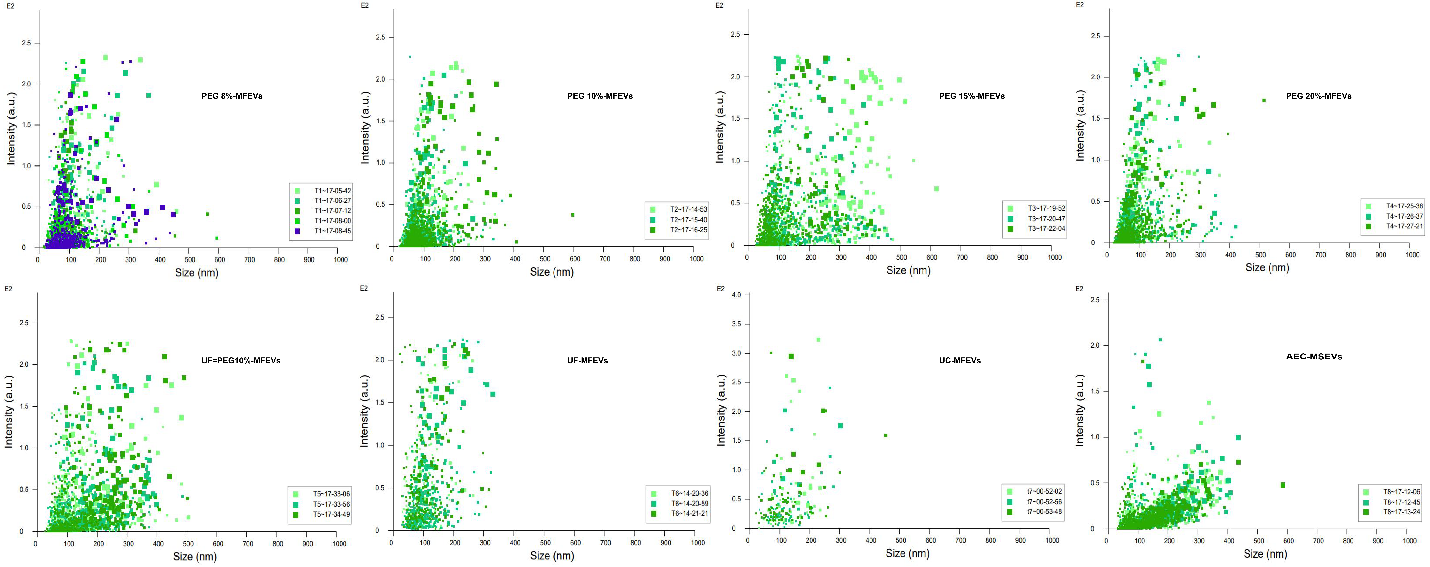


**Figure S3.** MFEV's protein concentrations with respect to the adopted isolation methods.


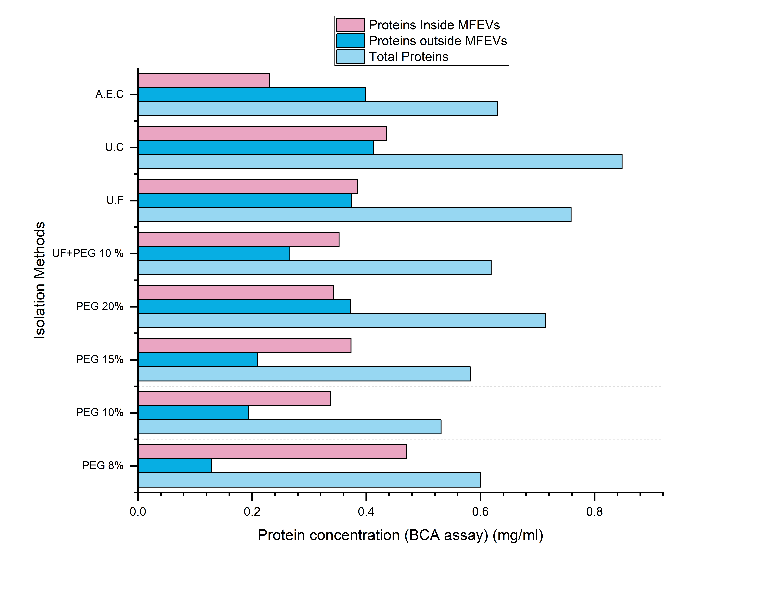


**Figure S4**. MFEV's nucleic acid concentrations with respect to the adopted isolation methods.


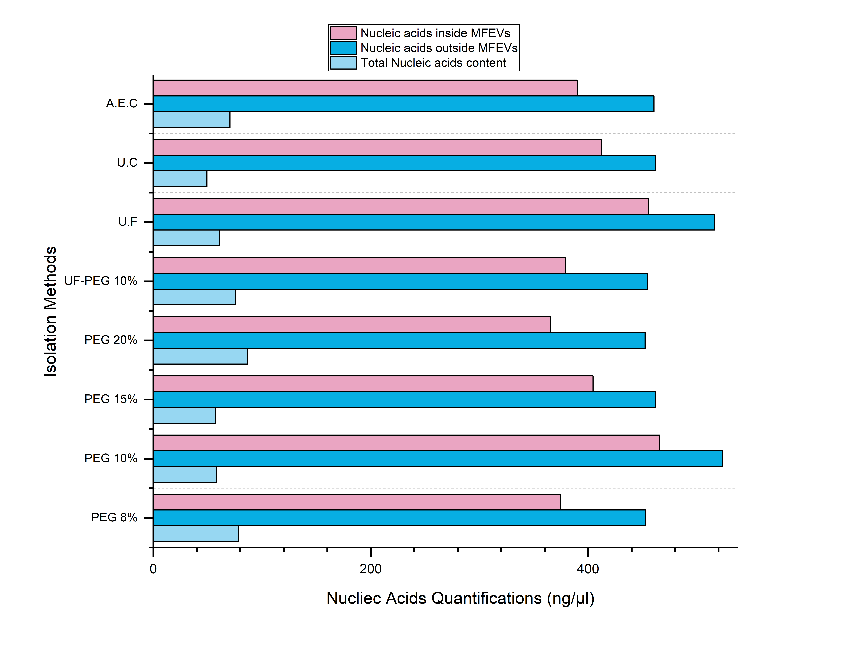


**Figure S5.** Monk fruit cells and cell cultures.


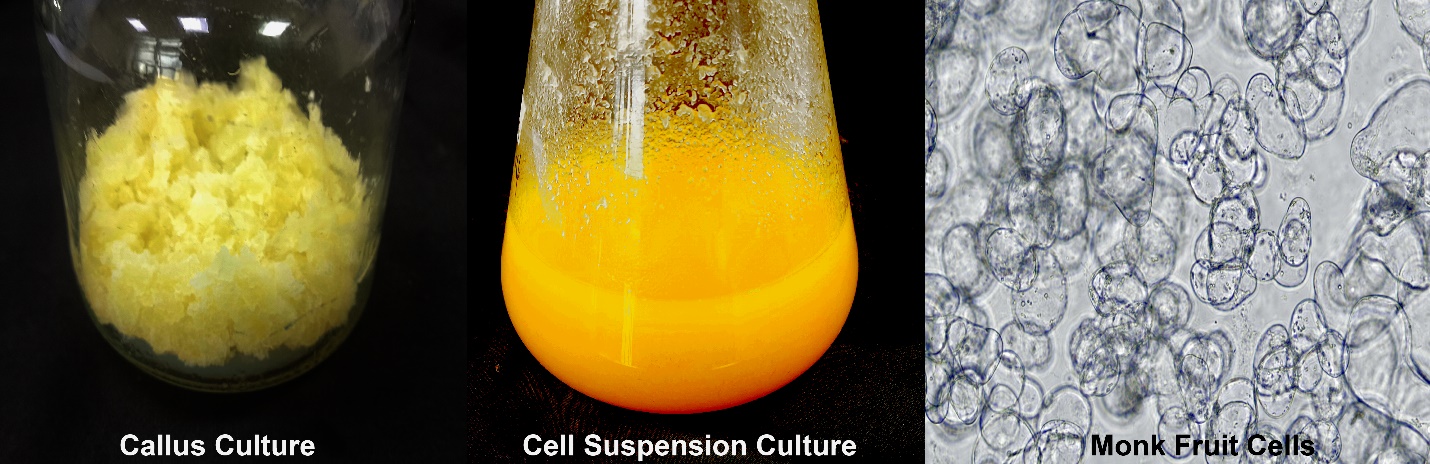


**Figure S6.** MFEVs quantitative bio-dispersal timeline for CHO and HEK-293 cell lines.


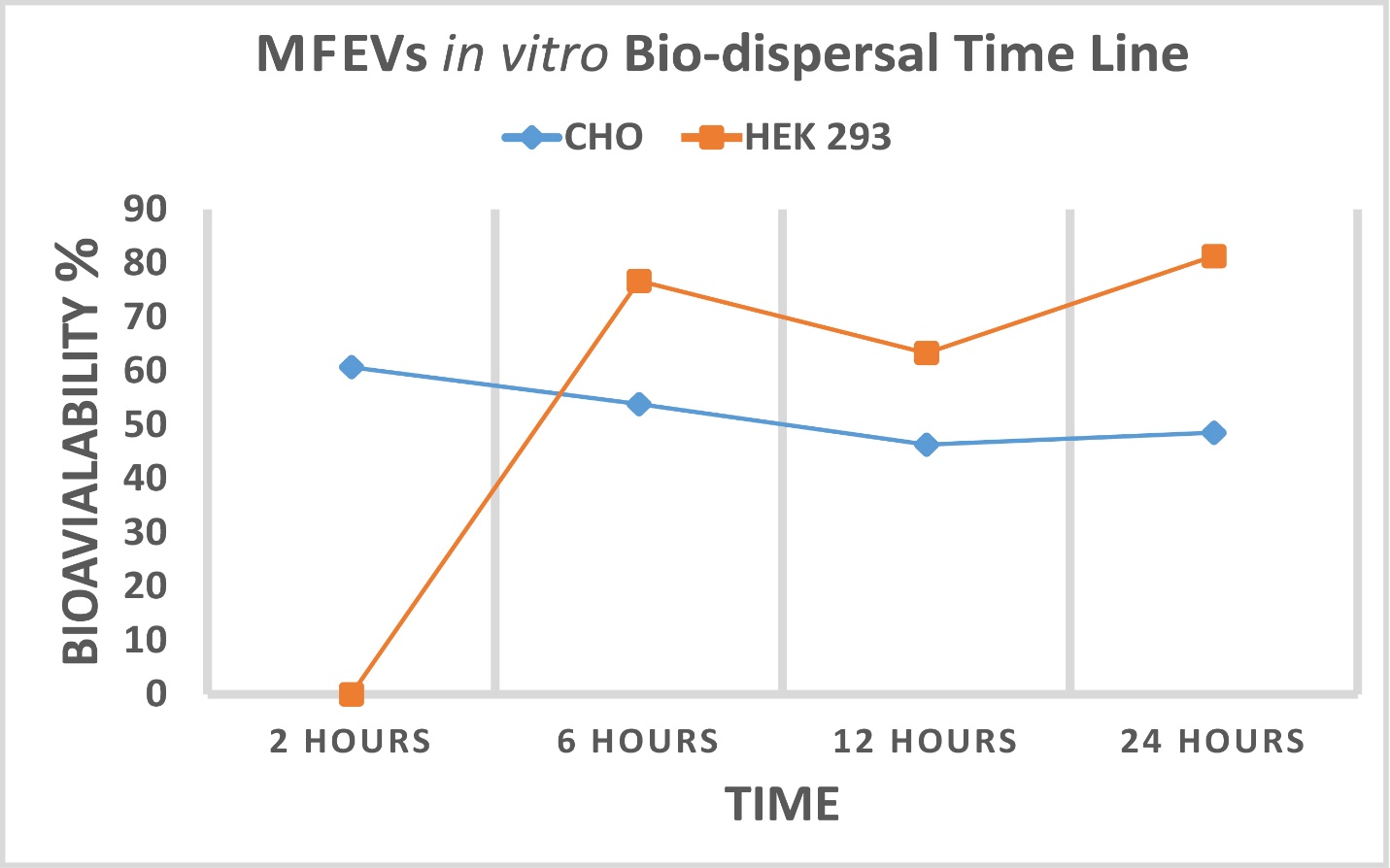


**Figure S7.** ExoOrb analyzed dataset 1 visualizations.

1. **Bar Plot of EVs Scores**


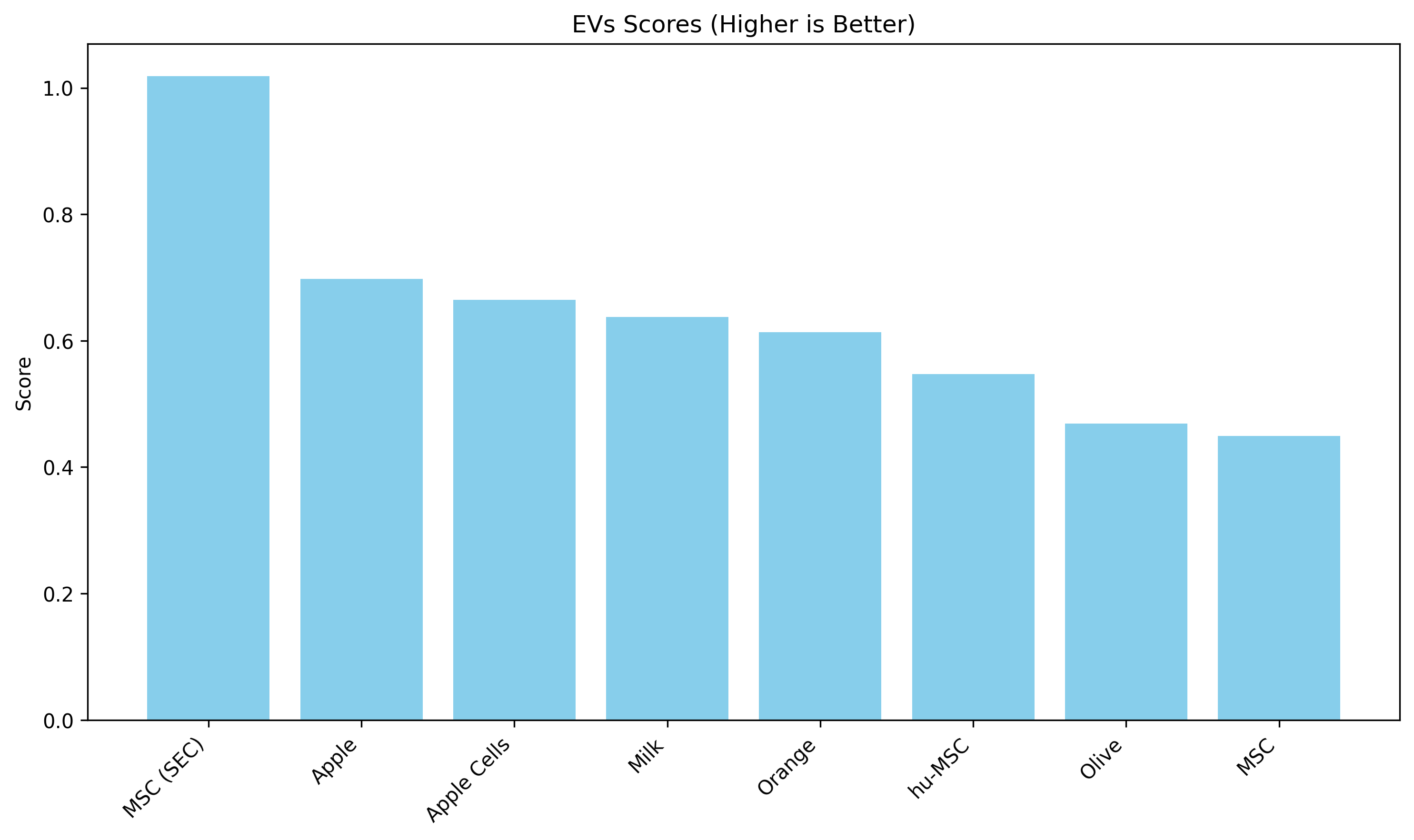


1. **Heatmap of Normalized Factors**


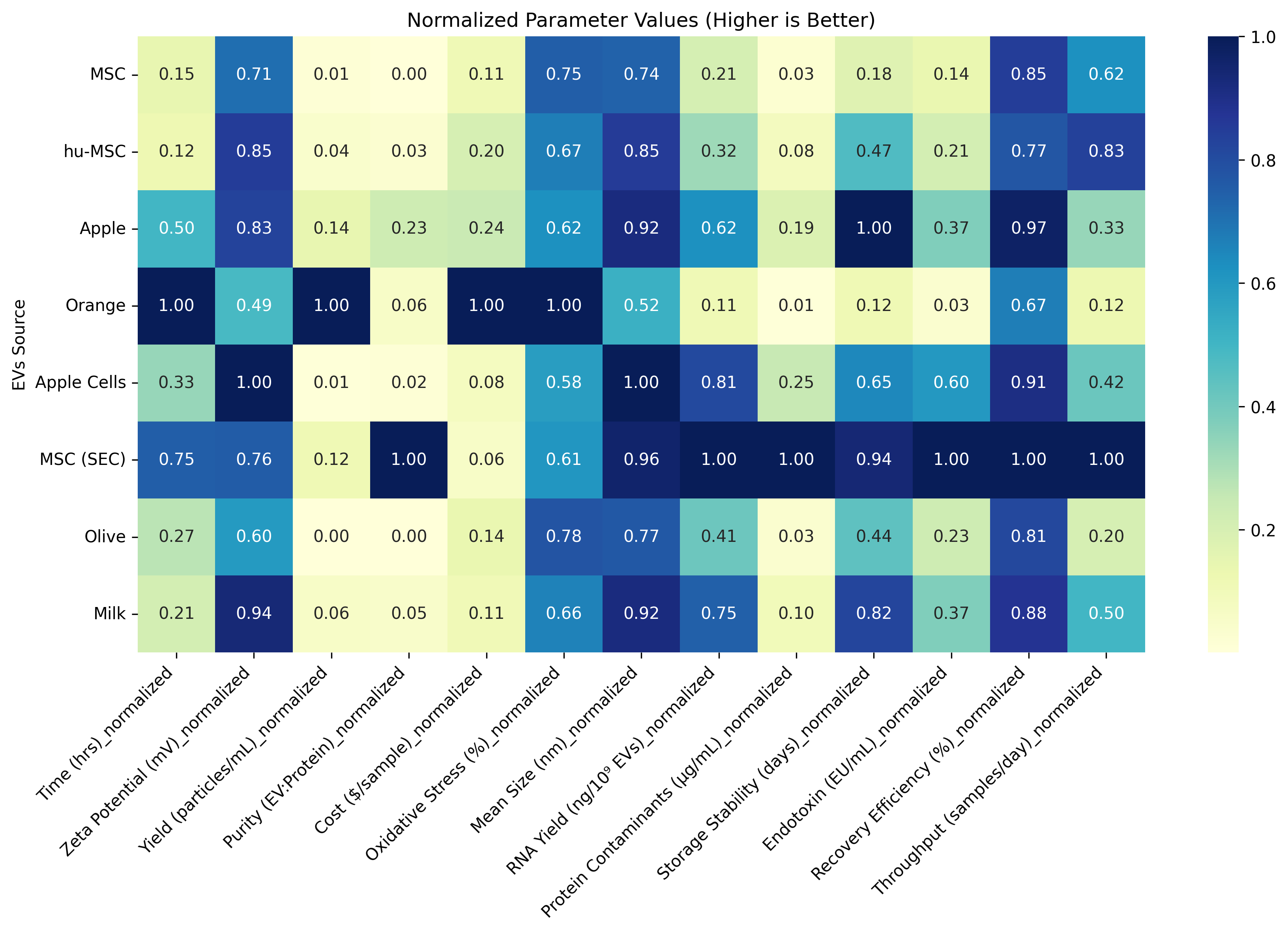


1. **Top EVs Radar Chart**


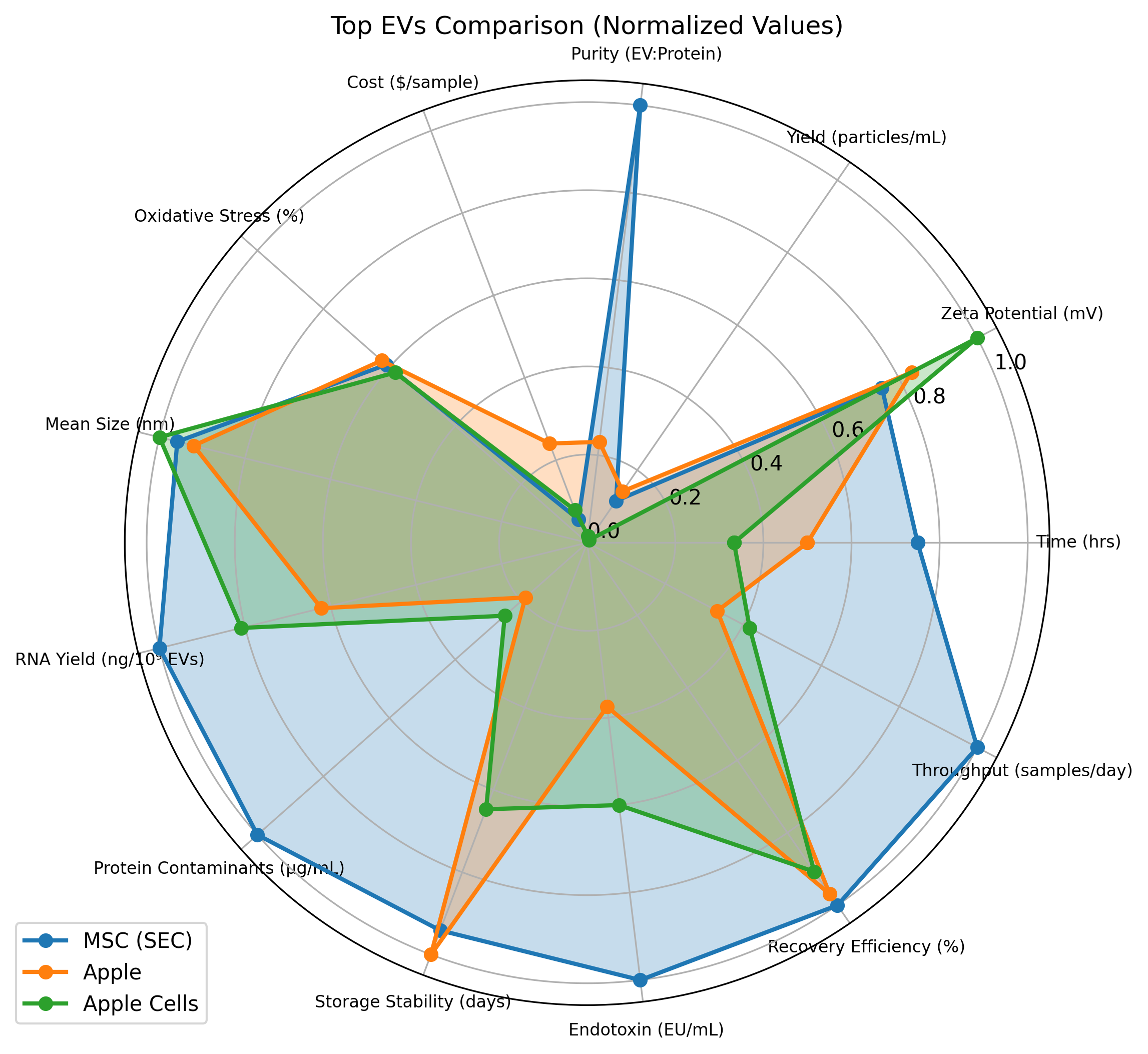


1. **3D Scatter Plot**
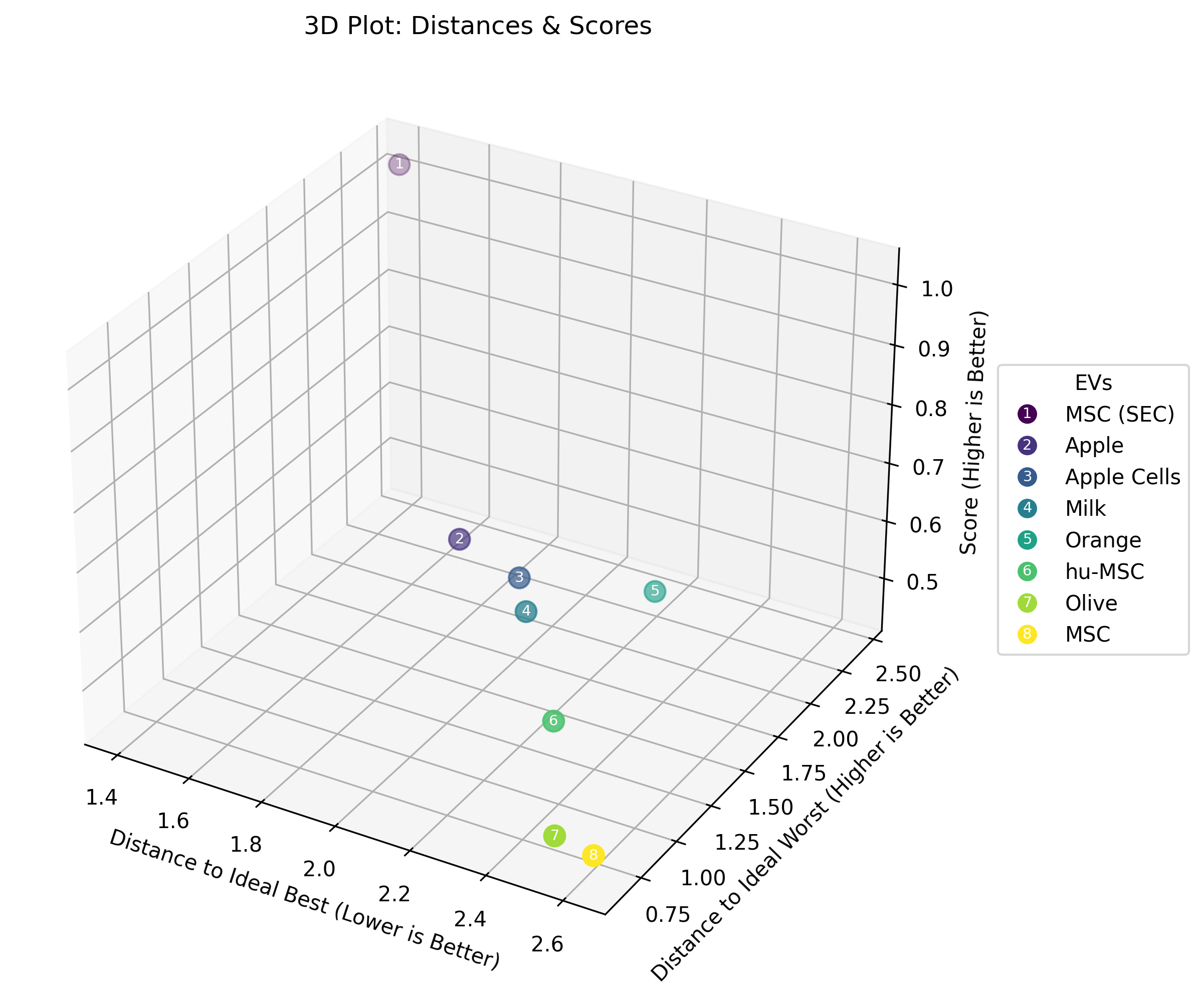


**Figure S8.** ExoOrb analyzed dataset 2 visualizations.

1. **Bar Plot of EVs Scores**


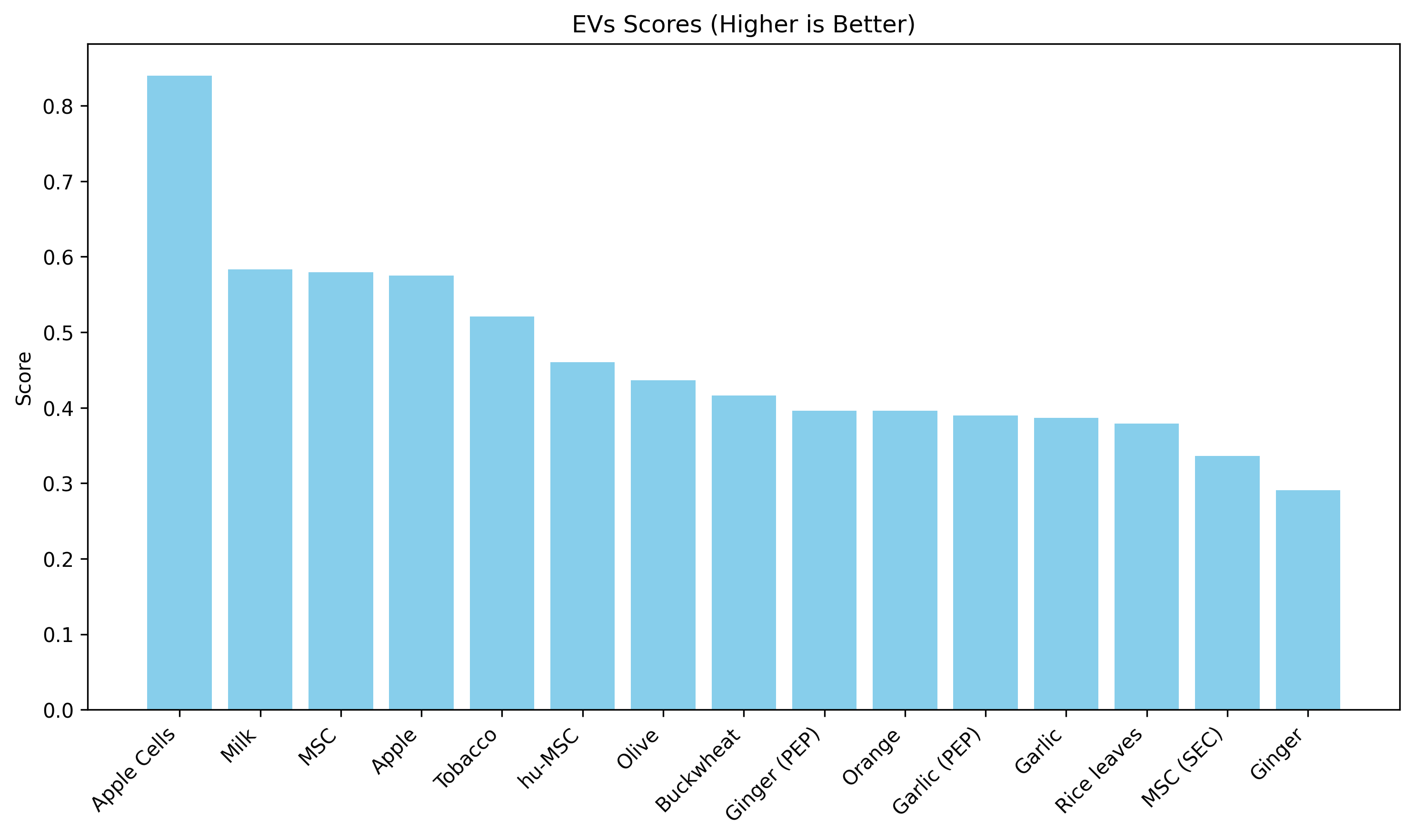


1. **Heatmap of Normalized Factors**


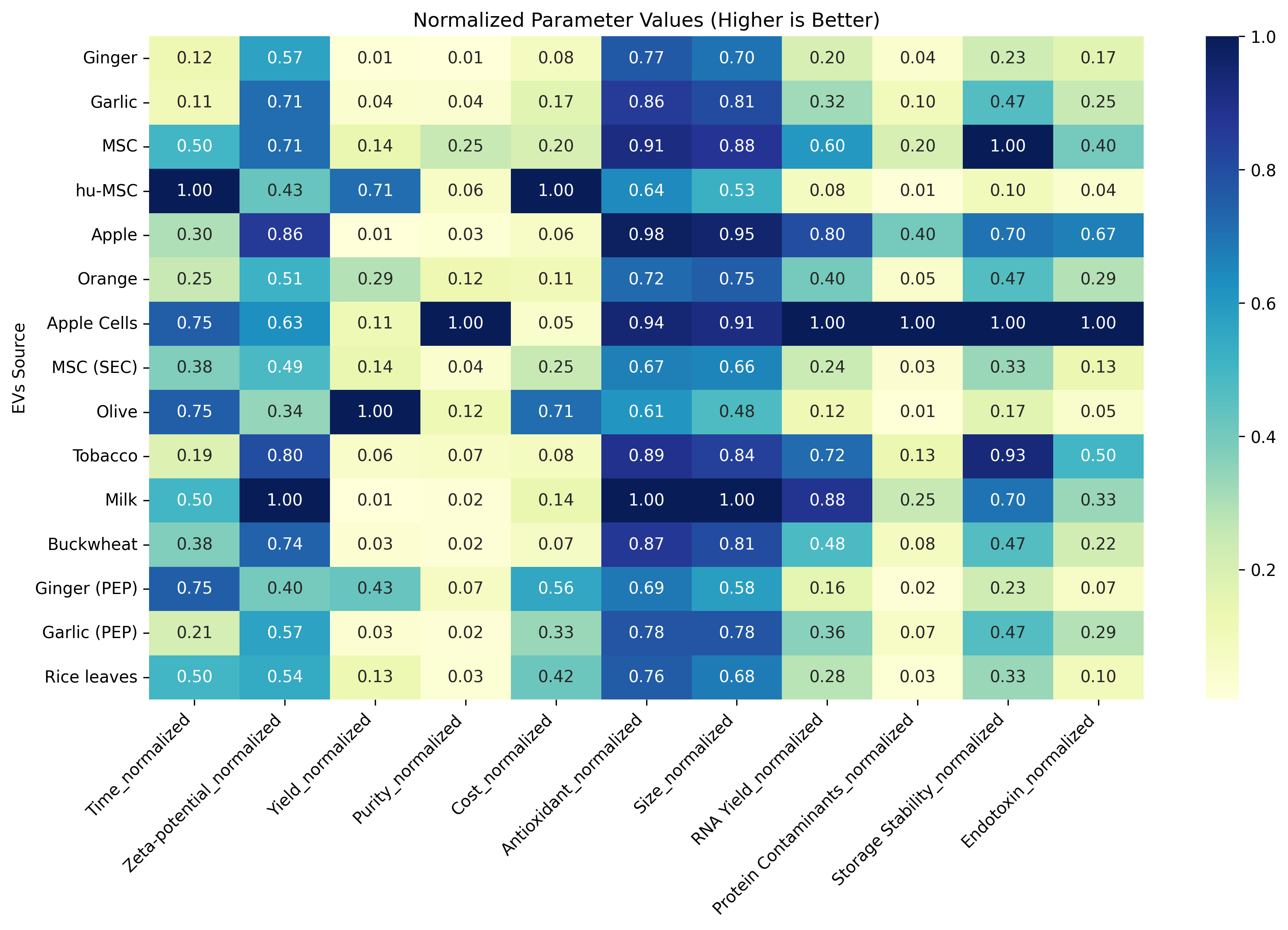


1. **Top EVs Radar Chart**


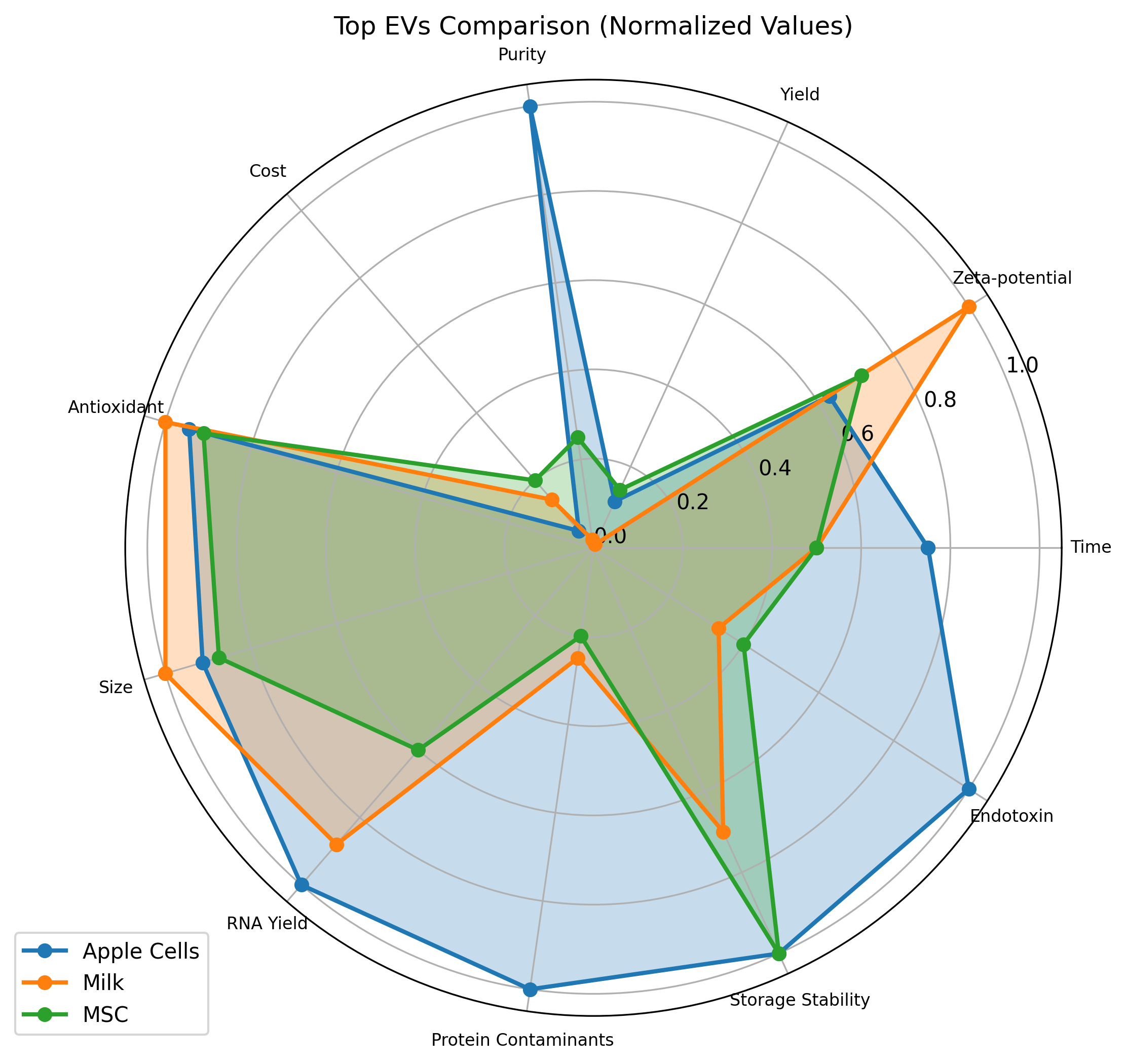


1. **3D Scatter Plot**
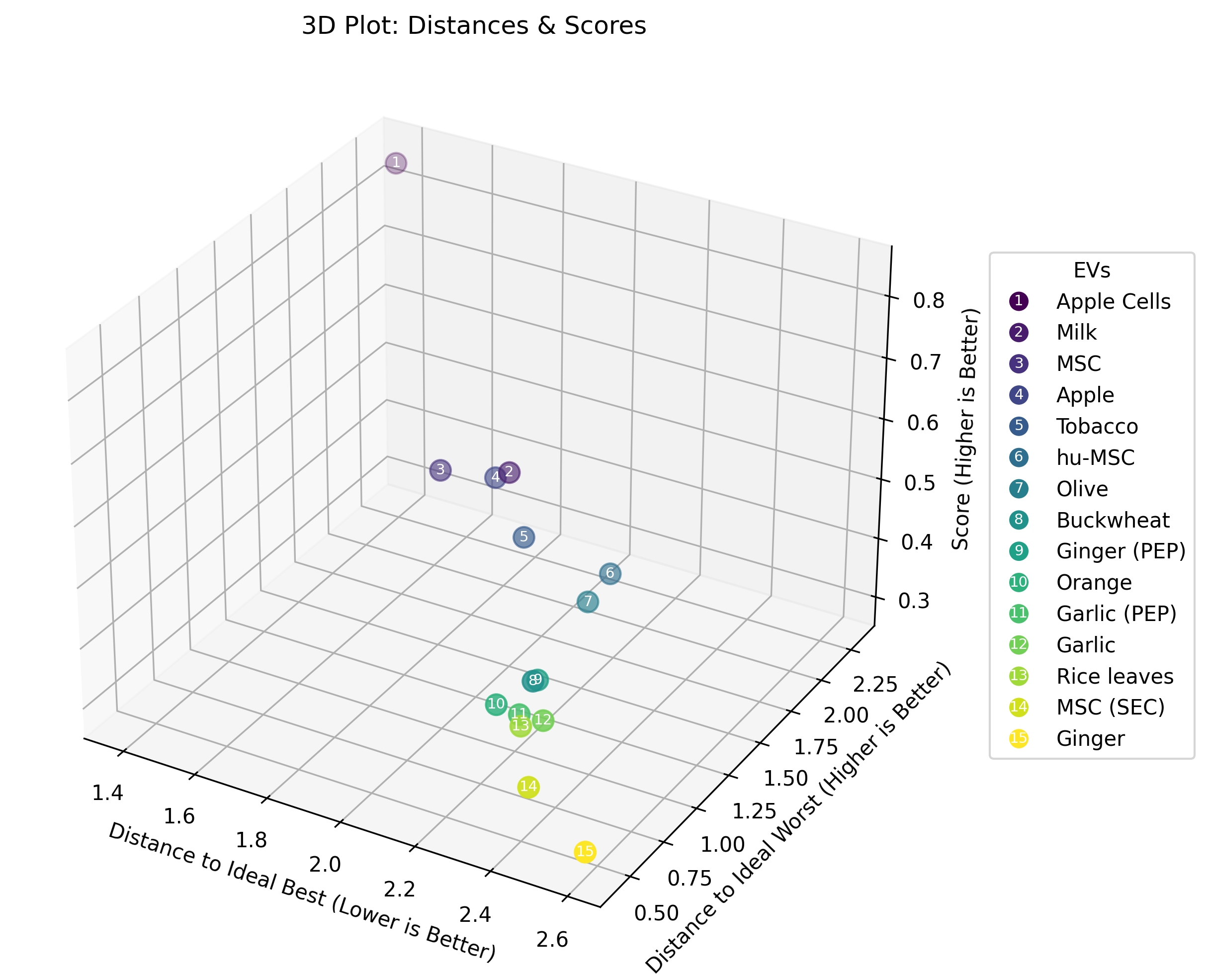


**Fig. S9. Customization of weightage for real dataset**


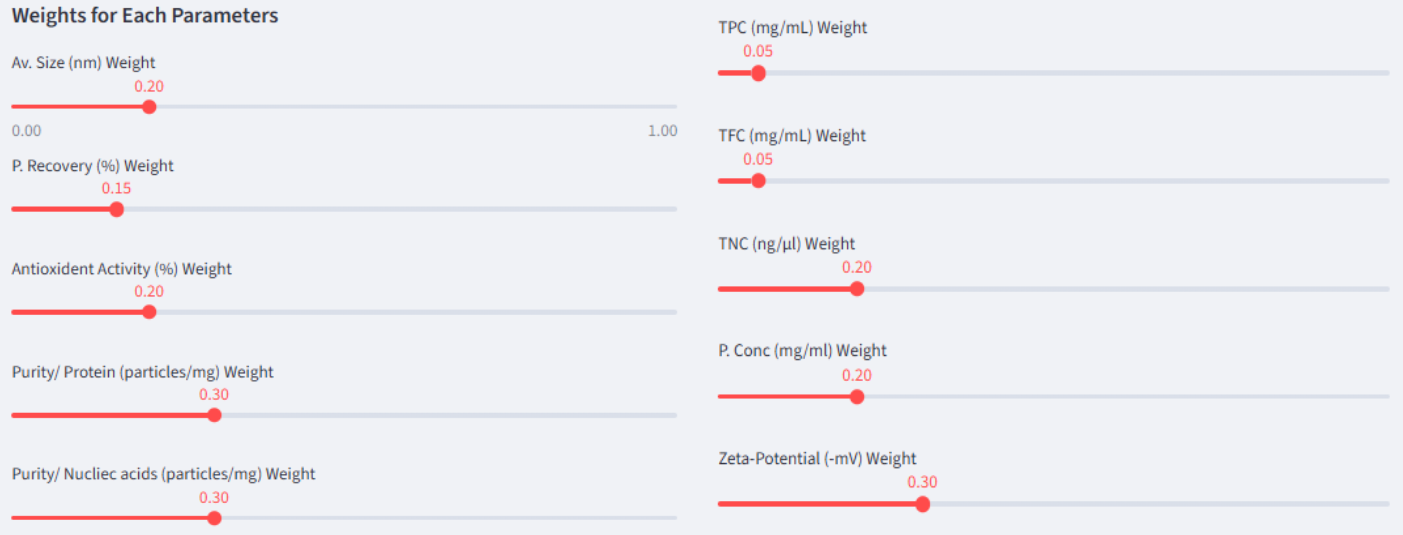


**Fig S10. ExoOrb’s customized weightage sensitivity analysis for a real dataset**

**(A)** EVs' overall score; **(B)** Factor normalization; **(C)** Radar chart top 3 EVs factor-based comparison; **(D)** 3D visualization plot presenting the EVs potential therapeutic rankings.


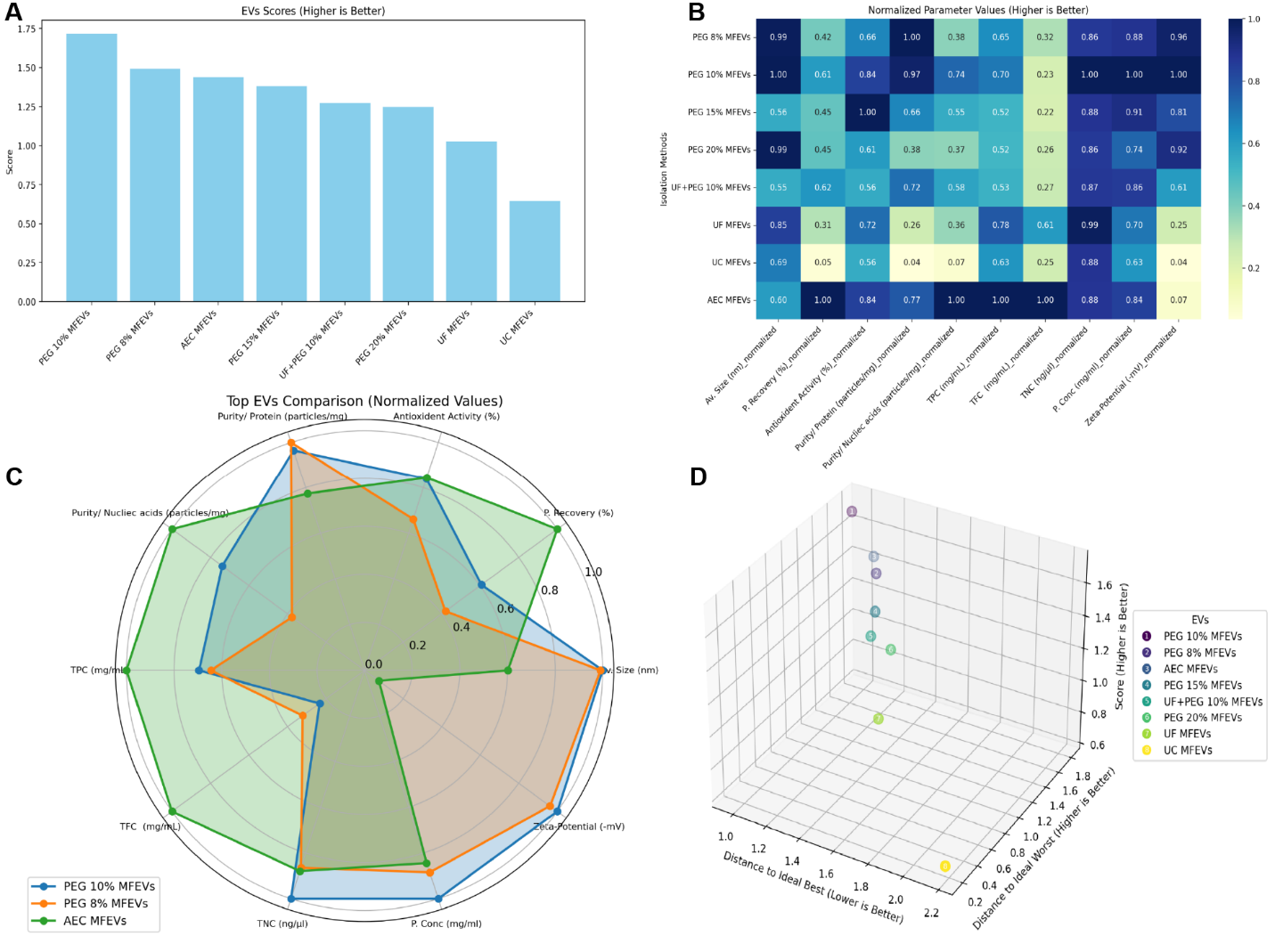

Supplement: Supplementary file 1 — Supplementary material [file mmc1.docx]
