# Supplementary material for: ExoOrb: A novel visual and analytical system for therapeutic extracellular vesicles metrics
Source: Comput Struct Biotechnol J. 2025 Nov 19;27:5289–306. doi: 10.1016/j.csbj.2025.11.038 (PMC12681852; doi:10.1016/j.csbj.2025.11.038)

EVs Comparison and Ranking Report

Generated on: 2025-07-18 03:41:48

Number of EVs compared: 15

# Parameters Classification

Parameters Maximized: Yield, Purity, Antioxidant, RNA Yield, Storage Stability

Parameters Minimized: Time, Zeta-potential, Cost, Size, Protein Contaminants, Endotoxin

# Parameters Weights

| Factor | Weight |
| --- | --- |
| Time | 0.10 |
| Zeta-potential | 0.10 |
| Yield | 0.10 |
| Purity | 0.10 |
| Cost | 0.10 |
| Antioxidant | 0.10 |
| Size | 0.10 |
| RNA Yield | 0.10 |
| Protein Contaminants | 0.10 |
| Storage Stability | 0.10 |
| Endotoxin | 0.10 |

# EVs Rankings

| Name | Score | Rank |
| --- | --- | --- |
| Apple Cells | 0.8400 | 1 |
| Milk | 0.5834 | 2 |
| MSC | 0.5793 | 3 |
| Apple | 0.5751 | 4 |
| Tobacco | 0.5210 | 5 |
| hu-MSC | 0.4605 | 6 |
| Olive | 0.4362 | 7 |
| Buckwheat | 0.4161 | 8 |
| Ginger (PEP) | 0.3961 | 9 |
| Orange | 0.3961 | 10 |
| Garlic (PEP) | 0.3899 | 11 |
| Garlic | 0.3868 | 12 |
| Rice leaves | 0.3788 | 13 |
| MSC (SEC) | 0.3358 | 14 |
| Ginger | 0.2906 | 15 |

# Input Data

| EVs | Time | Zeta-potential | Yield | Purity | Cost | Antioxidant | Size | RNA Yield | Protein Contaminants | Storage Stability | Endotoxin |
| --- | --- | --- | --- | --- | --- | --- | --- | --- | --- | --- | --- |
| Ginger | 6.0 | -20.0 | 10000000000.0 | 200000000.0 | 6000.0 | 69.0 | 150.0 | 5.0 | 50.0 | 7.0 | 1.2 |
| Garlic | 7.0 | -25.0 | 30000000000.0 | 1500000000.0 | 3000.0 | 77.0 | 130.0 | 8.0 | 20.0 | 14.0 | 0.8 |
| MSC | 1.5 | -25.0 | 100000000000.0 | 10000000000.0 | 2500.0 | 82.0 | 120.0 | 15.0 | 10.0 | 30.0 | 0.5 |
| hu-MSC | 0.75 | -15.0 | 500000000000.0 | 2500000000.0 | 500.0 | 58.0 | 200.0 | 2.0 | 200.0 | 3.0 | 5.0 |
| Apple | 2.5 | -30.0 | 5000000000.0 | 1000000000.0 | 8000.0 | 88.0 | 110.0 | 20.0 | 5.0 | 21.0 | 0.3 |
| Orange | 3.0 | -18.0 | 200000000000.0 | 5000000000.0 | 4500.0 | 65.0 | 140.0 | 10.0 | 40.0 | 14.0 | 0.7 |
| Apple Cells | 1.0 | -22.0 | 80000000000.0 | 40000000000.0 | 10000.0 | 85.0 | 115.0 | 25.0 | 2.0 | 30.0 | 0.2 |
| MSC (SEC) | 2.0 | -17.0 | 100000000000.0 | 1666666666.6666667 | 2000.0 | 60.0 | 160.0 | 6.0 | 60.0 | 10.0 | 1.5 |
| Olive | 1.0 | -12.0 | 700000000000.0 | 4666666666.666667 | 700.0 | 55.0 | 220.0 | 3.0 | 150.0 | 5.0 | 4.0 |
| Tobacco | 4.0 | -28.0 | 40000000000.0 | 2666666666.6666665 | 6000.0 | 80.0 | 125.0 | 18.0 | 15.0 | 28.0 | 0.4 |
| Milk | 1.5 | -35.0 | 6000000000.0 | 750000000.0 | 3500.0 | 90.0 | 105.0 | 22.0 | 8.0 | 21.0 | 0.6 |
| Buckwheat | 2.0 | -26.0 | 20000000000.0 | 800000000.0 | 7000.0 | 78.0 | 130.0 | 12.0 | 25.0 | 14.0 | 0.9 |
| Ginger (PEP) | 1.0 | -14.0 | 300000000000.0 | 3000000000.0 | 900.0 | 62.0 | 180.0 | 4.0 | 100.0 | 7.0 | 3.0 |
| Garlic (PEP) | 3.5 | -20.0 | 20000000000.0 | 666666666.6666666 | 1500.0 | 70.0 | 135.0 | 9.0 | 30.0 | 14.0 | 0.7 |
| Rice leaves | 1.5 | -19.0 | 90000000000.0 | 1125000000.0 | 1200.0 | 68.0 | 155.0 | 7.0 | 80.0 | 10.0 | 2.0 |

# Visualizations

## Bar Plot of Scores


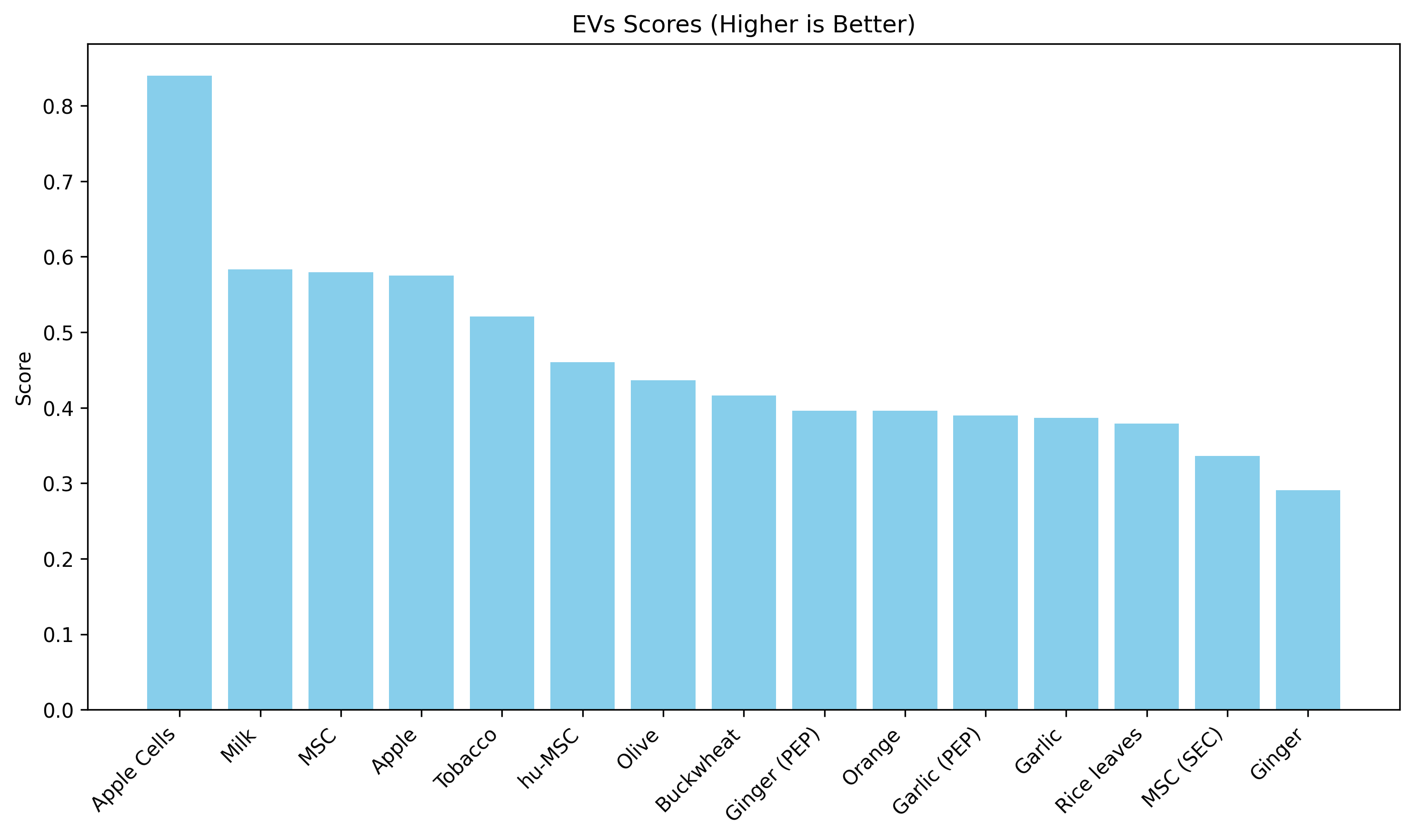


## Heatmap of Normalized Parameters


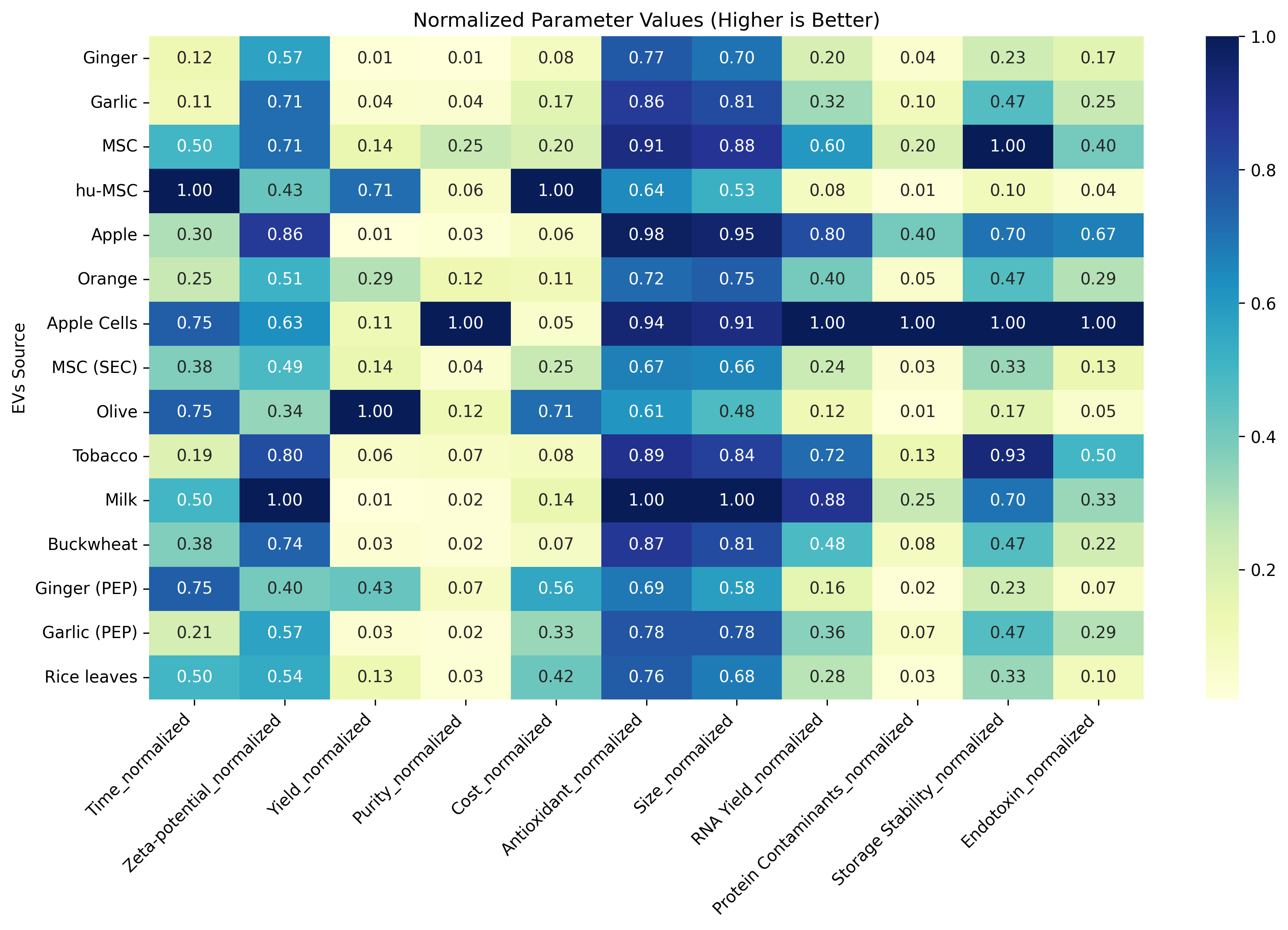


## Top EVs Radar Chart


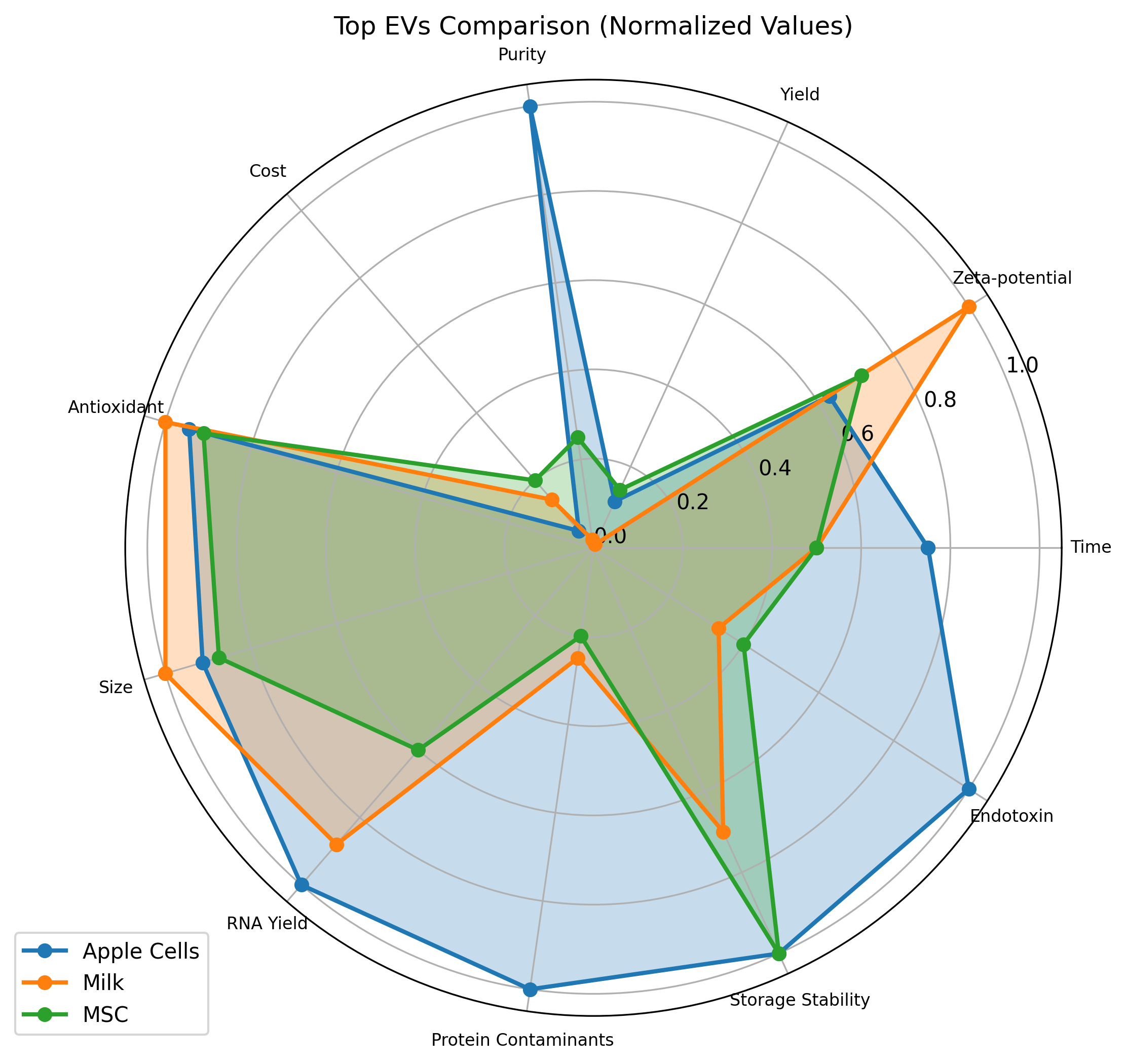


## 3D Scatter Plot


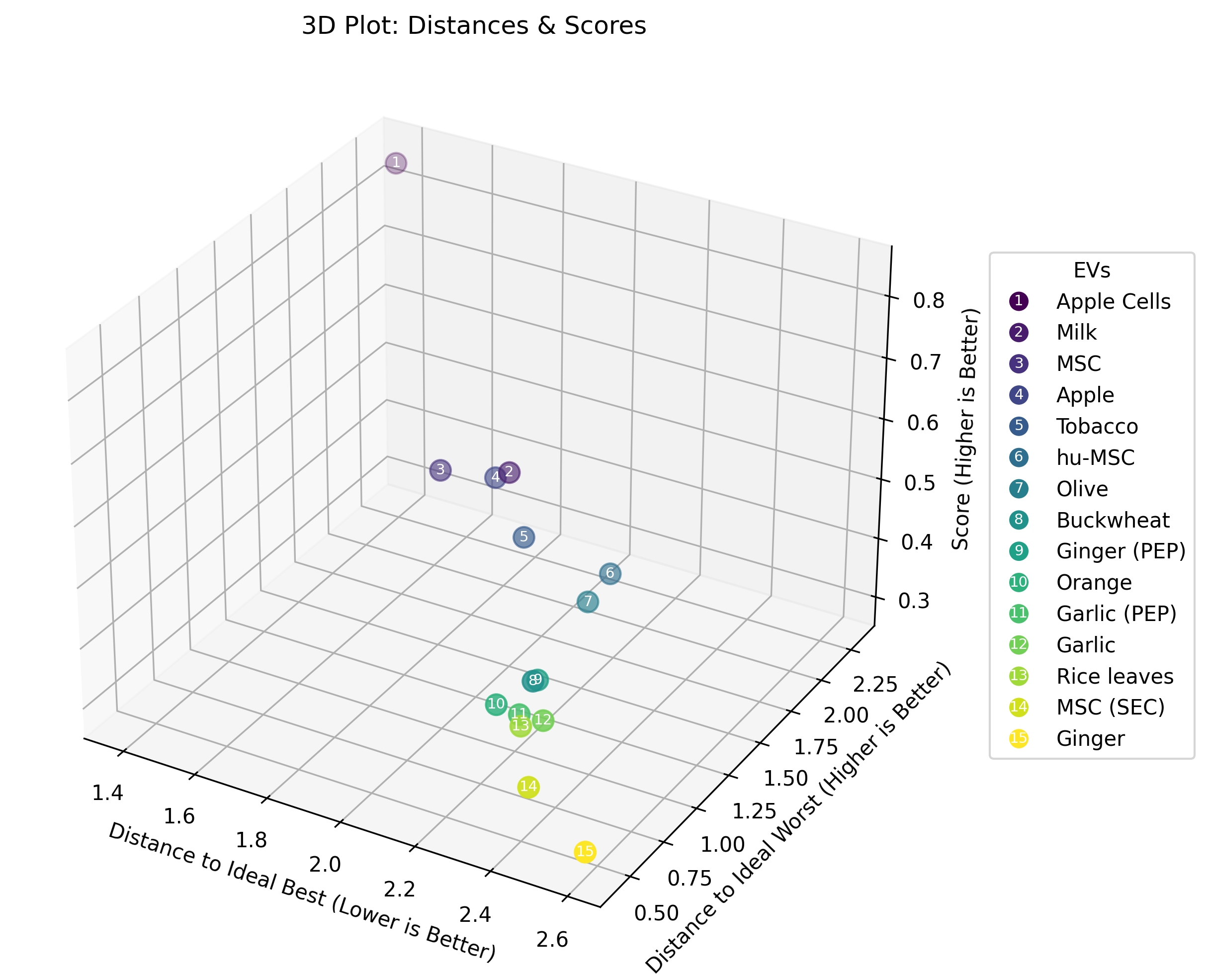

Supplement: Supplementary file 5 — Supplementary material [file mmc5.docx]
