# Supplementary material for: ExoOrb: A novel visual and analytical system for therapeutic extracellular vesicles metrics
Source: Comput Struct Biotechnol J. 2025 Nov 19;27:5289–306. doi: 10.1016/j.csbj.2025.11.038 (PMC12681852; doi:10.1016/j.csbj.2025.11.038)

EVs Comparison and Ranking Report

Generated on: 2025-07-18 03:43:55

Number of EVs compared: 8

# Parameters Classification

Parameters Maximized: Yield (particles/mL), Purity (EV:Protein), RNA Yield (ng/10⁹ EVs), Storage Stability (days), Recovery Efficiency (%)

Parameters Minimized: Time (hrs), Zeta Potential (mV), Cost ($/sample), Oxidative Stress (%), Mean Size (nm), Protein Contaminants (μg/mL), Endotoxin (EU/mL), Throughput (samples/day)

# Parameters Weights

| Factor | Weight |
| --- | --- |
| Time (hrs) | 0.10 |
| Zeta Potential (mV) | 0.10 |
| Yield (particles/mL) | 0.10 |
| Purity (EV:Protein) | 0.10 |
| Cost ($/sample) | 0.10 |
| Oxidative Stress (%) | 0.10 |
| Mean Size (nm) | 0.10 |
| RNA Yield (ng/10⁹ EVs) | 0.10 |
| Protein Contaminants (μg/mL) | 0.10 |
| Storage Stability (days) | 0.10 |
| Endotoxin (EU/mL) | 0.10 |
| Recovery Efficiency (%) | 0.10 |
| Throughput (samples/day) | 0.10 |

# EVs Rankings

| Name | Score | Rank |
| --- | --- | --- |
| MSC (SEC) | 1.0186 | 1 |
| Apple | 0.6974 | 2 |
| Apple Cells | 0.6647 | 3 |
| Milk | 0.6372 | 4 |
| Orange | 0.6131 | 5 |
| hu-MSC | 0.5469 | 6 |
| Olive | 0.4689 | 7 |
| MSC | 0.4491 | 8 |

# Input Data

| EVs | Time (hrs) | Zeta Potential (mV) | Yield (particles/mL) | Purity (EV:Protein) | Cost ($/sample) | Oxidative Stress (%) | Mean Size (nm) | RNA Yield (ng/10⁹ EVs) | Protein Contaminants (μg/mL) | Storage Stability (days) | Endotoxin (EU/mL) | Recovery Efficiency (%) | Throughput (samples/day) |
| --- | --- | --- | --- | --- | --- | --- | --- | --- | --- | --- | --- | --- | --- |
| MSC | 6.2 | -21.5 | 10500000000.0 | 190909090.9090909 | 5800.0 | 71.0 | 155.0 | 5.2 | 55.0 | 6.0 | 1.1 | 82.0 | 8.0 |
| hu-MSC | 7.5 | -25.8 | 31000000000.0 | 1722222222.2222223 | 3200.0 | 79.0 | 135.0 | 8.1 | 18.0 | 16.0 | 0.7 | 75.0 | 6.0 |
| Apple | 1.8 | -25.2 | 102000000000.0 | 12750000000.0 | 2700.0 | 85.0 | 125.0 | 15.6 | 8.0 | 34.0 | 0.4 | 94.0 | 15.0 |
| Orange | 0.9 | -14.7 | 720000000000.0 | 3272727272.7272725 | 650.0 | 53.0 | 220.0 | 2.8 | 220.0 | 4.0 | 4.5 | 65.0 | 40.0 |
| Apple Cells | 2.7 | -30.3 | 5100000000.0 | 850000000.0 | 8200.0 | 91.0 | 115.0 | 20.3 | 6.0 | 22.0 | 0.25 | 88.0 | 12.0 |
| MSC (SEC) | 1.2 | -22.9 | 83000000000.0 | 55333333333.333336 | 11500.0 | 87.0 | 120.0 | 25.1 | 1.5 | 32.0 | 0.15 | 97.0 | 5.0 |
| Olive | 3.3 | -18.1 | 2150000000.0 | 47777777.777777776 | 4800.0 | 68.0 | 150.0 | 10.4 | 45.0 | 15.0 | 0.65 | 79.0 | 25.0 |
| Milk | 4.2 | -28.4 | 42500000000.0 | 2833333333.3333335 | 6000.0 | 80.0 | 125.0 | 18.7 | 15.0 | 28.0 | 0.4 | 85.0 | 10.0 |

# Visualizations

## Bar Plot of Scores


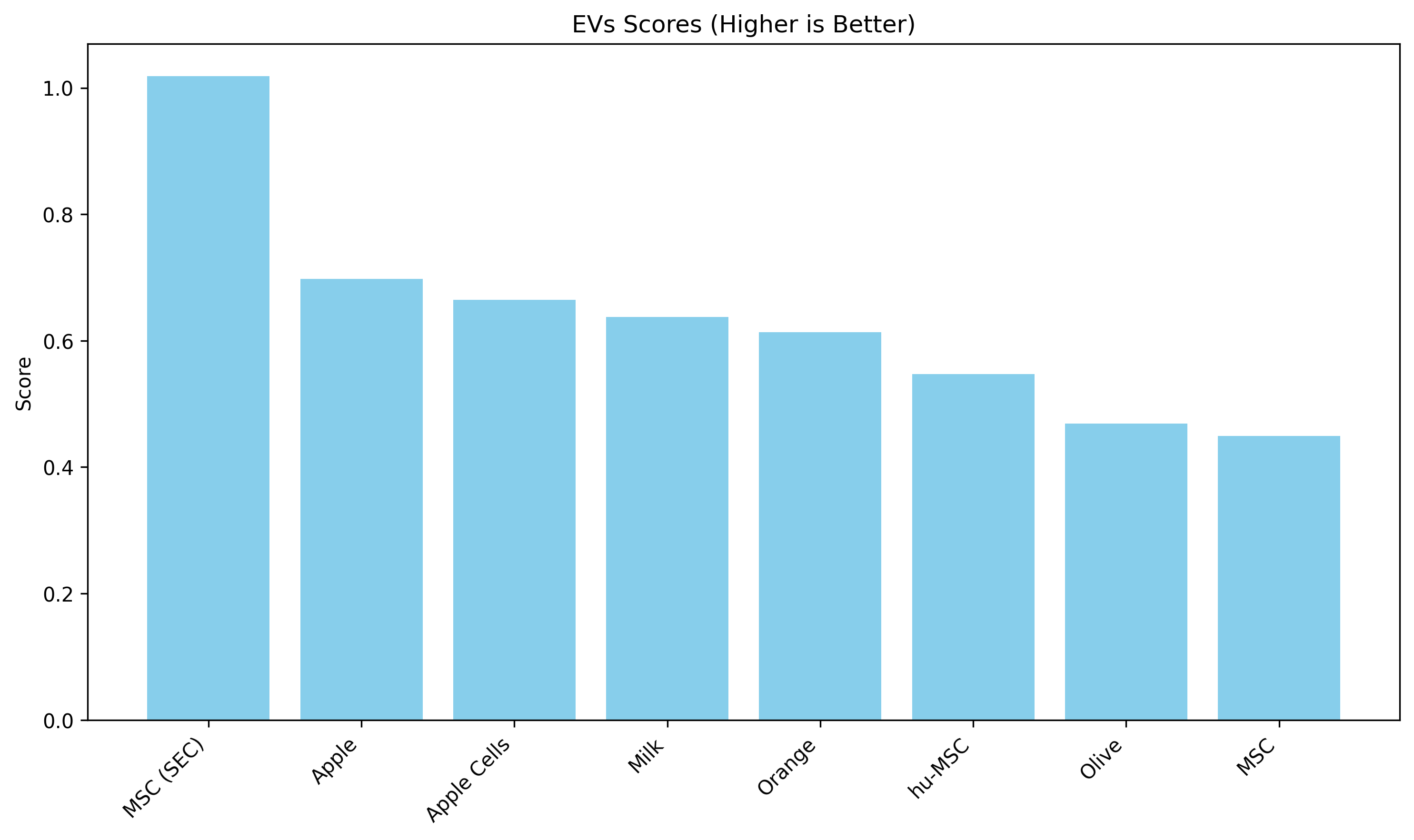


## Heatmap of Normalized Parameters


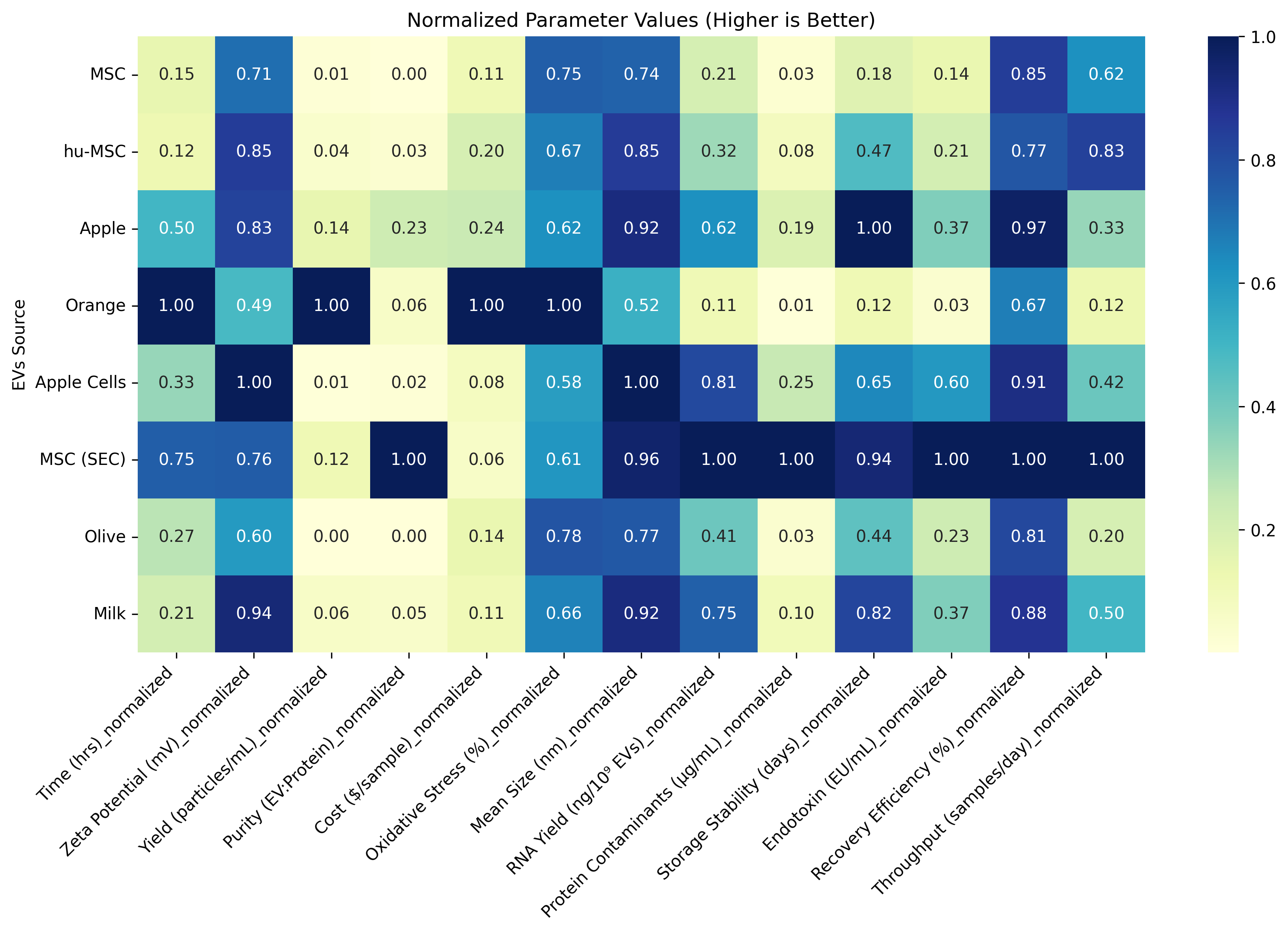


## Top EVs Radar Chart


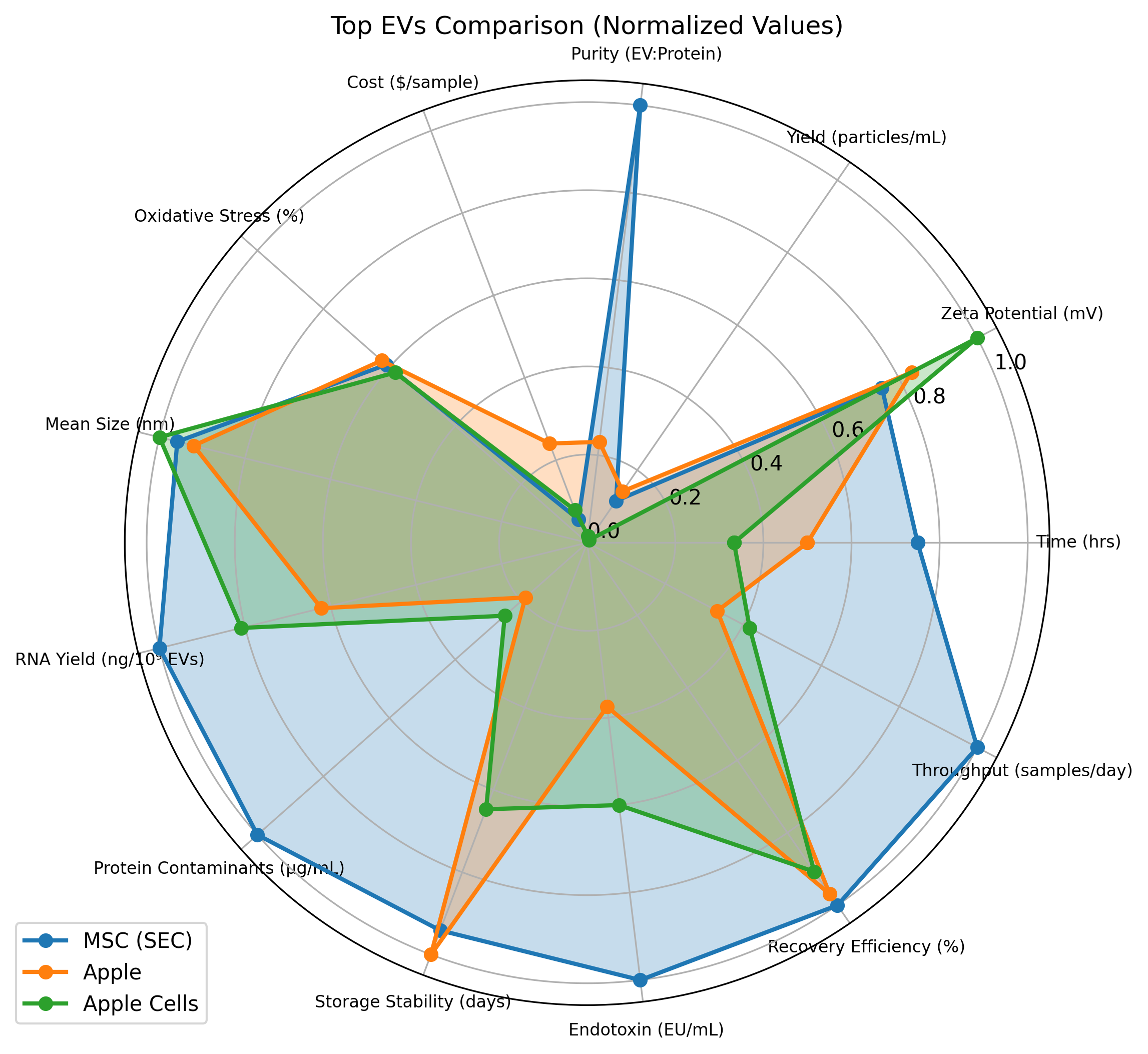


## 3D Scatter Plot


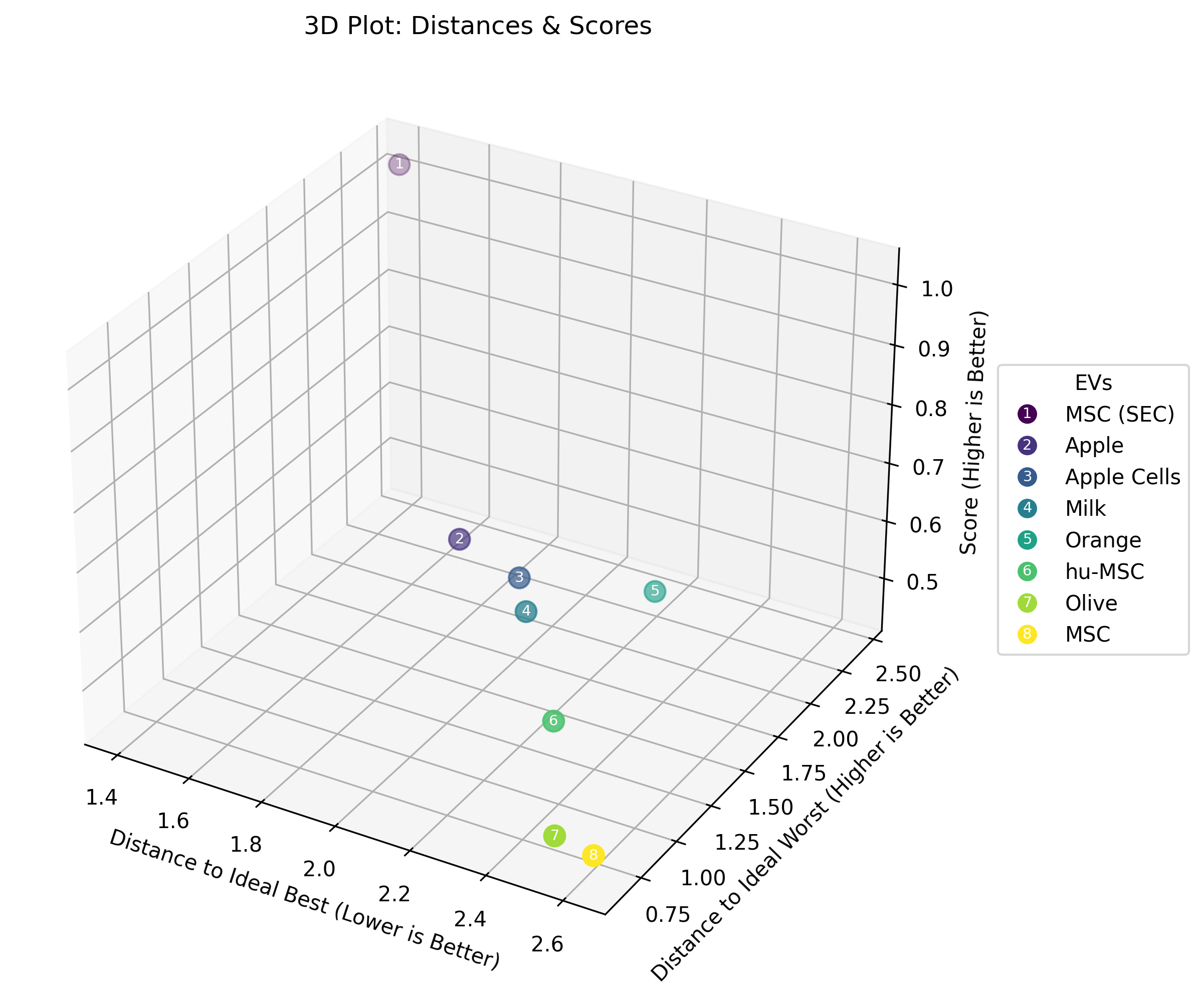

Supplement: Supplementary file 6 — Supplementary material [file mmc6.docx]
