# Supplementary figures and images for: ExoOrb: A novel visual and analytical system for therapeutic extracellular vesicles metrics
Source: Comput Struct Biotechnol J. 2025 Nov 19;27:5289–306. doi: 10.1016/j.csbj.2025.11.038 (PMC12681852; doi:10.1016/j.csbj.2025.11.038)

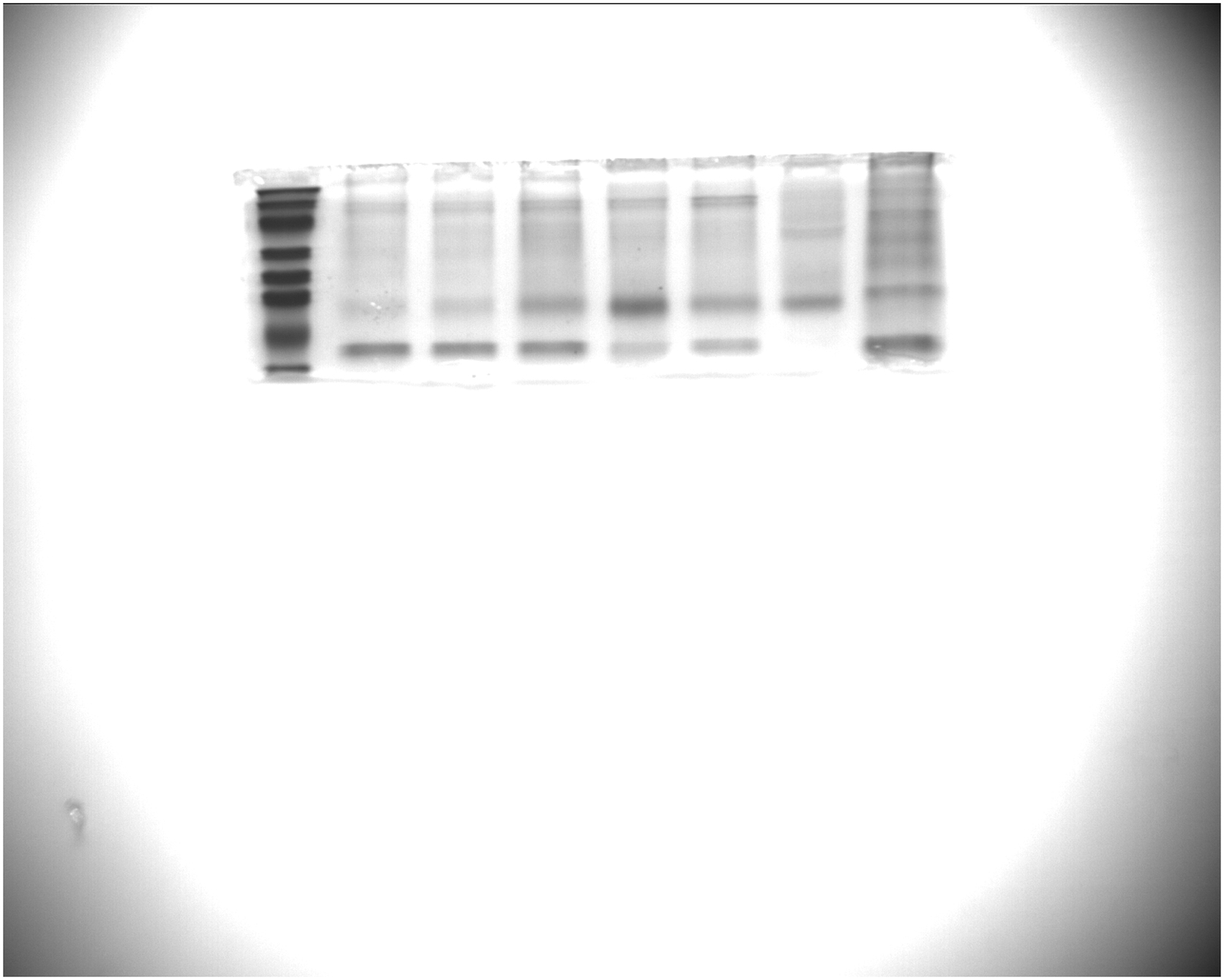

Supplement: Supplementary file 6 — Supplementary material [file mmc7.jpg]

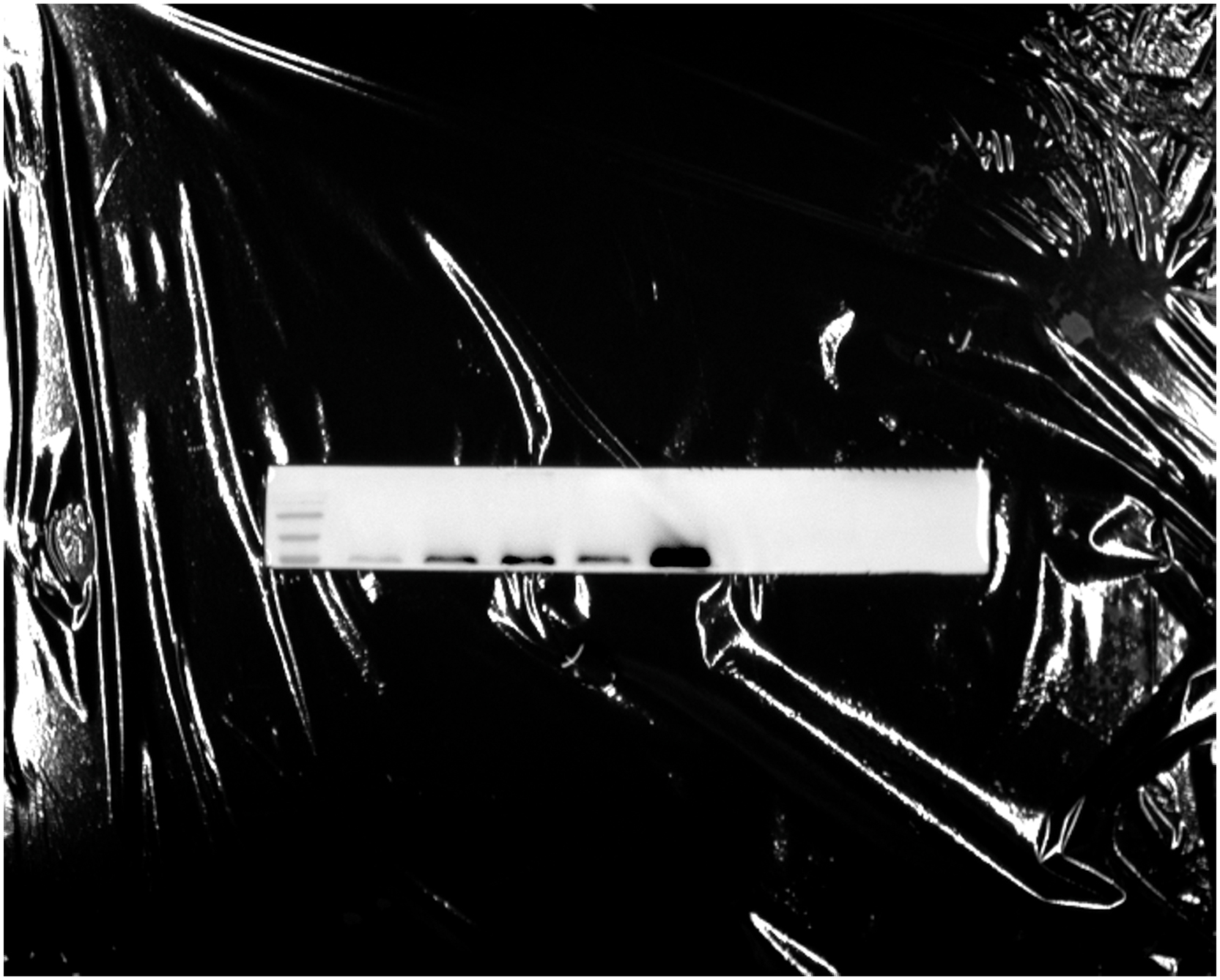

Supplement: Supplementary file 6 — Supplementary material [file mmc8.jpg]

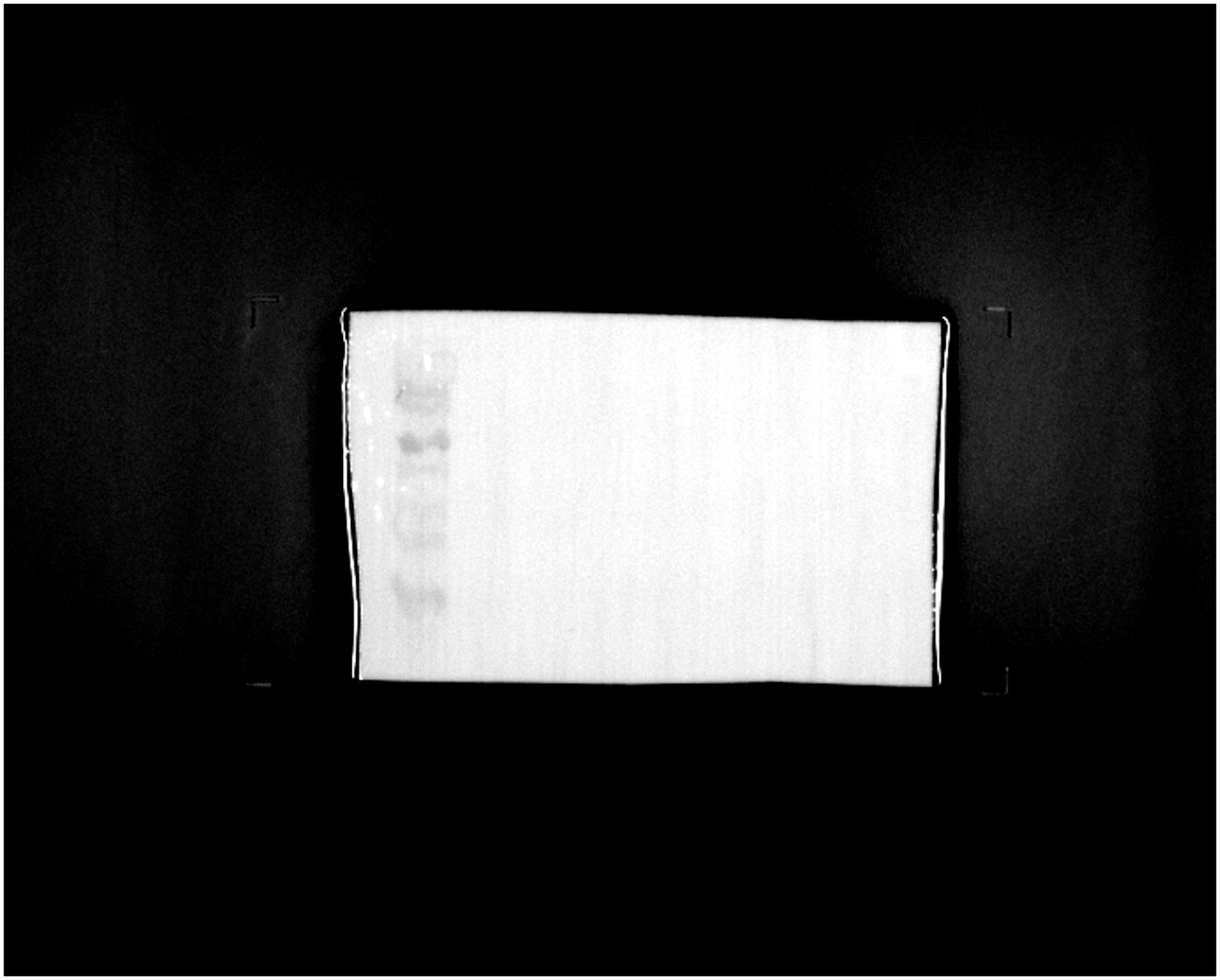

Supplement: Supplementary file 6 — Supplementary material [file mmc9.jpg]

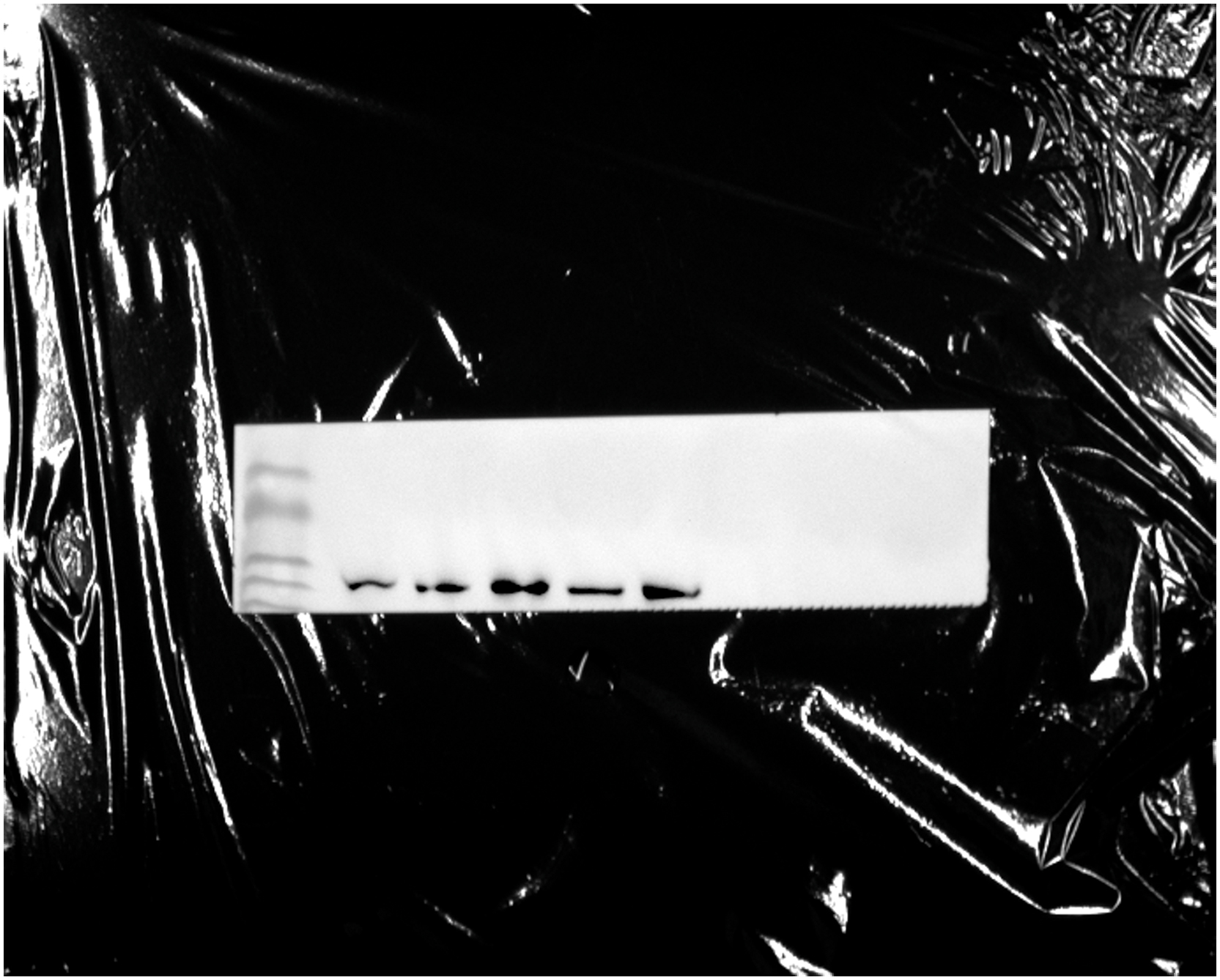

Supplement: Supplementary file 6 — Supplementary material [file mmc10.jpg]
